# An anti-ACVR1 antibody exacerbates heterotopic ossification by fibro-adipogenic progenitors in fibrodysplasia ossificans progressiva mice

**DOI:** 10.1172/JCI153795

**Published:** 2022-06-15

**Authors:** John B. Lees-Shepard, Sean J. Stoessel, Julian T. Chandler, Keith Bouchard, Patricia Bento, Lorraine N. Apuzzo, Parvathi M. Devarakonda, Jeffrey W. Hunter, David J. Goldhamer

**Affiliations:** 1 Department of Molecular & Cell Biology, University of Connecticut Stem Cell Institute, University of Connecticut, Storrs, Connecticut, USA.; 2 Alexion Pharmaceuticals Inc., New Haven, Connecticut, USA.

**Keywords:** Bone Biology, Stem cells, Adult stem cells, Bone disease, Skeletal muscle

## Abstract

Fibrodysplasia ossificans progressiva (FOP) is a rare genetic disease characterized by progressive and catastrophic heterotopic ossification (HO) of skeletal muscle and associated soft tissues. FOP is caused by dominantly acting mutations in the gene encoding the bone morphogenetic protein (BMP) type I receptor, ACVR1 (ALK2), the most prevalent of which results in an arginine to histidine substitution at position 206 (ACVR1[R206H]). The fundamental pathological consequence of FOP-causing ACVR1 receptor mutations is to enable activin A to initiate canonical BMP signaling in fibro-adipogenic progenitors (FAPs), which drives HO. We developed a monoclonal blocking antibody (JAB0505) against the extracellular domain of ACVR1 and tested its effect on HO in 2 independent FOP mouse models. Although JAB0505 inhibited BMP-dependent gene expression in wild-type and ACVR1(R206H)-overexpressing cell lines, JAB0505 treatment profoundly exacerbated injury-induced HO. JAB0505-treated mice exhibited multiple, distinct foci of heterotopic lesions, suggesting an atypically broad anatomical domain of FAP recruitment to endochondral ossification. This was accompanied by dysregulated FAP population growth and an abnormally sustained immunological reaction following muscle injury. JAB0505 drove injury-induced HO in the absence of activin A, indicating that JAB0505 has receptor agonist activity. These data raise serious safety and efficacy concerns for the use of bivalent anti-ACVR1 antibodies to treat patients with FOP.

## Graphical abstract

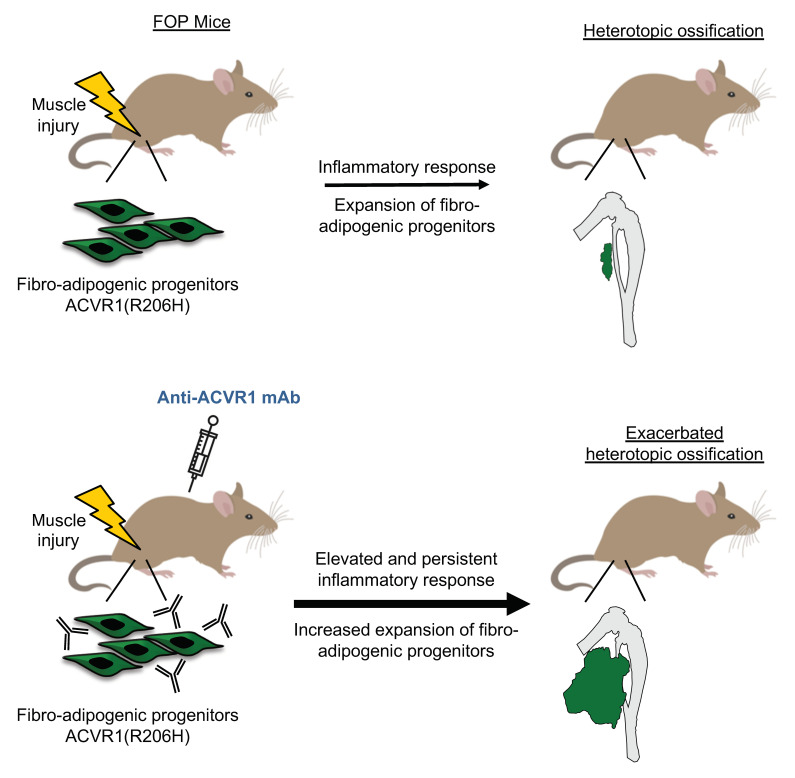

## Introduction

Fibrodysplasia ossificans progressiva (FOP) is a rare, autosomal dominant, genetic disease of progressive heterotopic ossification (HO) that primarily affects skeletal muscle and associated connective tissues. The cumulative effect of HO, which typically begins in early childhood (1), leads to progressive immobility and shortened lifespan (2). The most common FOP mutation in *Acvr1* results in an arginine to histidine amino acid substitution at position 206 in the intracellular glycine-serine domain of ACVR1 (ACVR1[R206H]; ref. 3), which renders this bone morphogenetic protein (BMP) receptor responsive to activin ligands (4–7) and hypersensitive to select BMP ligands (8–11). While the physiological relevance of hypersensitivity to BMP ligands is unclear, blocking antibodies against activin A are highly effective at inhibiting HO in preclinical mouse models of FOP, and a clinical trial is underway to evaluate this strategy (ClinicalTrials.gov NCT03188666). Here, we address whether an antibody directed against the extracellular domain (ECD) of ACVR1(R206H) that interferes with ligand-dependent receptor activation would inhibit HO and constitute a potential therapeutic approach for FOP. We present the unexpected finding that an anti-ACVR1 antibody (JAB0505) that effectively blocks ligand-dependent osteogenic reporter gene expression in cultured C2C12 cells profoundly exacerbates HO in FOP mouse models. As our previous work and that of others indicate that fibro-adipogenic progenitors (FAPs) are a key causative cell type in FOP (6, 12, 13), we addressed how antibody treatment affects BMP signaling, expansion, and differentiation of ACVR1(R206H)-expressing FAPs (R206H-FAPs). We demonstrate that JAB0505 acts as a weak agonist of ACVR1(R206H) in FAPs and can activate osteogenic signaling, even in the absence of activin A. Treatment of FOP mice with JAB0505 altered the expansion, recruitment, and osteogenic differentiation kinetics of R206H-FAPs and caused a dysregulated immunological response to muscle injury, all of which likely conspired to dramatically enhance HO. Recently, independent studies have confirmed extreme worsening of HO with anti-ACVR1 antibodies (14). Together, these data indicate that blockade of ligand-receptor interactions by the use of bivalent anti-ACVR1 monoclonal antibodies (mAbs) is not a viable therapeutic approach for FOP.

## Results

### Characterization of the anti-ACVR1 mAb JAB0505.

**Figure 1:**
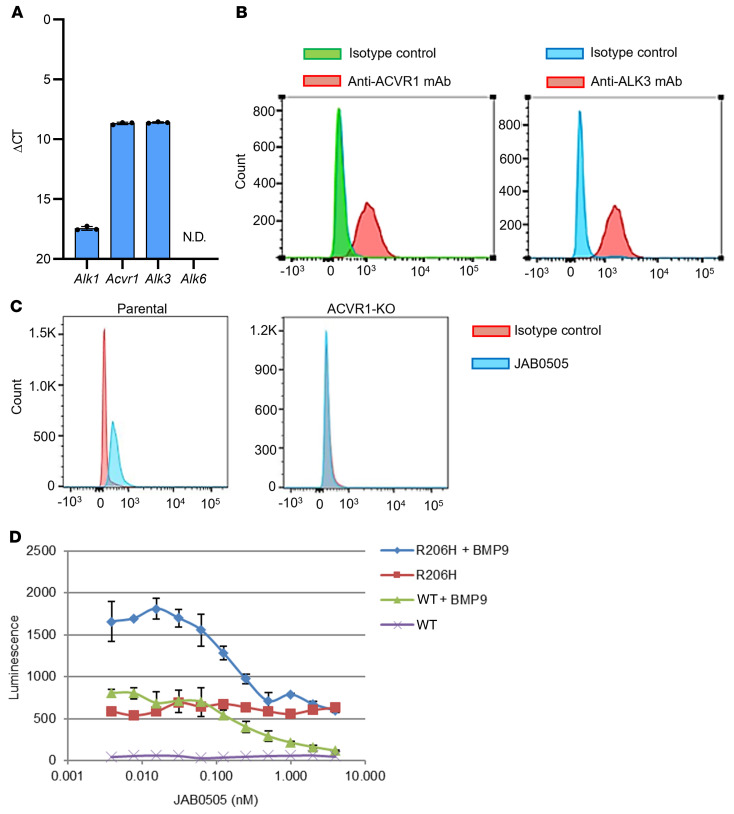
JAB0505 is an ACVR1-blocking mAb. (**A**) Mouse C2C12 myoblast cells express similar levels of *Acvr1* and *Alk3*, low levels of *Alk1*, and no detectable *Alk6*, as quantified by RT-qPCR (*n =* 3). ΔCT values were calculated using the average CT values of the internal controls, *Gapdh* or *Actb* (β-actin) (see Methods). Error bars represent ±SD. CT values >40 were considered not detected (N.D.). (**B**) Surface expression of ACVR1 and ALK3 on C2C12 cells, as detected by flow cytometry. (**C**) mAb JAB0505 binds to parental C2C12 cells, but not *Acvr1*-KO cells, as assessed by flow cytometry. (**D**) JAB0505 inhibits BMP9-induced signal activation in wild-type and ACVR1(R206H)-overexpressing C2C12 cells in a dose-dependent manner, as determined by quantification of BRE-luciferase activity (*n =* 3). Error bars represent ±SD.

A mouse anti-ACVR1 mAb was isolated from an immune-biased phage-display antibody library that was constructed from mice immunized with DNA encoding the ECD of ACVR1. This antibody was affinity matured by PCR mutagenesis of the complementarity determining regions (CDRs) to yield the antibody JAB0505 (Supplemental Figure 1; supplemental material available online with this article; https://doi.org/10.1172/JCI153795DS1). Affinity maturation improved binding to ACVR1 and inhibition of ligand-induced signaling in vitro (Supplemental Figure 2, A–C). The equilibrium dissociation constant (*K_D_*) of JAB0505 for binding to ACVR1 was determined to be approximately 2.5 × 10^–8^ M by surface plasmon resonance (Supplemental Figure 2A). Quantification of BMP type I receptor mRNA abundance in C2C12 cells by RT-qPCR revealed levels of *Alk3* (*Bmpr1a*) similar to those of *Acvr1* (*Alk2*), much lower levels of *Alk1*, and no detectable *Alk6* mRNA (Figure 1A). Cell surface expression of ACVR1 and ALK3 on C2C12 cells was confirmed by flow cytometry with receptor-specific antibodies (Figure 1B). JAB0505 does not recognize ALK3, as evidenced by the loss of cell surface binding of JAB0505 to ACVR1-knockout (ACVR1-KO) C2C12 cells (Figure 1C). The ability of JAB0505 to block ligand-dependent BMP signaling was assessed in wild-type and ACVR1(R206H)-transfected C2C12 cells by quantifying activity of the Id1-luciferase reporter, BRE-Luc, a well-established transcriptional readout of BMP signaling activity (15). JAB0505 completely blocked BMP6-, BMP7-, BMP9-, and BMP10-induced luciferase activity in the wild-type C2C12-BRE-Luc reporter cell line (Figure 1D and Supplemental Figure 2, C–F). In the ACVR1(R206H)-transfected C2C12-BRE-Luc reporter cell line, JAB0505 did not diminish luciferase activity in response to BMP2 or BMP4 (Supplemental Figure 2, G and H), which signal principally through ALK3 (16), but blocked hyperresponsive signaling to BMP9 (Figure 1D and Supplemental Figure 2B). Although BMP9 also signals via ALK1, the return to baseline with JAB0505 treatment, and the very low expression level of ALK1 in this cell line (Figure 1A), strongly suggests that the observed BMP9-dependent signaling is almost exclusively mediated by ACVR1. Baseline luciferase activity was substantially higher than that observed for wild-type reporter cells and unaffected by JAB0505 (Figure 1D), probably reflecting either weak, ligand-independent signaling due to overexpression of the mutant receptor, or differential responsiveness to ligands in the growth serum.

### JAB0505 dramatically exacerbates and prolongs HO in FOP mice.

**Figure 2:**
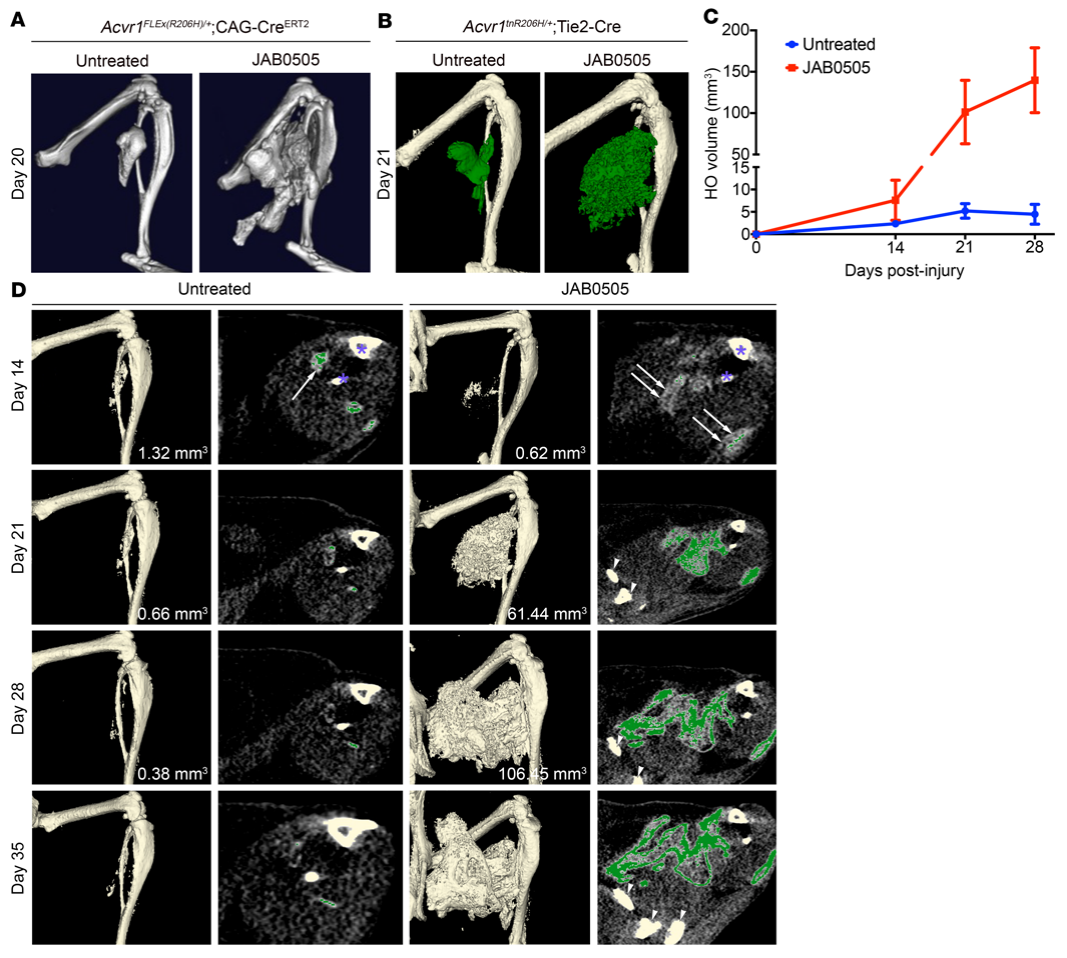
JAB0505 profoundly exacerbates HO and extends the period of lesional growth in FOP mice. (**A**) Representative μCT images of HO in *Acvr1^FLEx(R206H)/+^*; CAG-Cre^ERT2^ mice 20 days after cardiotoxin-induced injury of the gastrocnemius muscle (Untreated, *n =* 3; JAB0505, *n =* 4). (**B**) Representative μCT images of HO (pseudocolored green) in *Acvr1^tnR206H/+^*; Tie2-Cre mice 21 days after pinch injury of the gastrocnemius muscle (Untreated, *n =* 11; JAB0505, *n =* 10). (**C**) Quantification of HO volumes as a function of time after muscle pinch injury of *Acvr1^tnR206H/+^*; Tie2-Cre mice. Untreated, *n =* 11; JAB0505 (10 mg/kg), *n =* 6. Error bars represent ±SEM. ****P ≤ 0.0001 by 2-way ANOVA with Sidak’s multiple-comparison test. (**D**) Paired single transverse slice and 3D reconstructed μCT images of the distal hind limb of *Acvr1^tnR206H/+^*; Tie2-Cre mice at the indicated times after hind limb muscle pinch injury with and without administration of JAB0505. Mineralized bone in the slice images is pseudocolored green. Radio-opaque lesional tissue below the threshold set for quantification of mineralized bone (white arrows in day 14 slices) is extensive at day 14 in JAB0505-treated mice. Mineralized bone in day 14 slices is barely visible at this magnification. HO volumes are given for images prior to day 35. The tibia and fibula are labeled with asterisks in the day 14 slices. Pelvic bones present in day 21, 28, and 35 slices of JAB0505-treated mice are denoted with arrowheads. To avoid confusion with HO, the baculum present in some images was removed by segmentation.

We next tested the ability of JAB0505 to inhibit injury-induced HO in FOP mouse models. First, we used conditional *Acvr1^FLEx(R206H)/+^*; CAG-Cre^ERT2^ mice in which recombination of the *Acvr1^R206H^* allele is driven by the ubiquitously expressed CAG-Cre driver and is tamoxifen dependent. Following tamoxifen administration and a 5- to 7-day washout period, HO was induced by intramuscular injection of cardiotoxin into the gastrocnemius muscle. One day prior to cardiotoxin injection, JAB0505 was administered at 10 mg/kg by tail vein injection. The serum concentrations of JAB0505 are provided in Supplemental Figure 3. Surprisingly, JAB0505 dramatically exacerbated the formation of heterotopic bone, as revealed by μCT on day 14 (data not shown) and day 20 (Figure 2A) after injury. To further investigate the mechanism of exacerbated heterotopic bone formation, we tested the effects of JAB0505 in *Acvr1^tnR206H^* mice (6), in which the Tie2-Cre driver (17) was used to target *Acvr1^R206H^* expression to FAPs, a major cell of origin in HO (6, 12, 18). In agreement with the observations in globally recombined *Acvr1^FLEx(R206H)/+^*; CAG-Cre^ERT2^ mice, a single i.p. injection of 10 mg/kg JAB0505 at the time of muscle pinch injury profoundly exacerbated HO in *Acvr1^tnR206H/+^*; Tie2-Cre FOP mice (Figure 2, B and C). Neither uninjured FOP mice nor control mice lacking either Tie2-Cre or the *Acvr1^tnR206H^* allele developed HO in response to JAB0505 (data not shown).

μCT imaging of untreated *Acvr1^tnR206H/+^*; Tie2-Cre mice at weekly intervals from 2 to 5 weeks after injury showed that mineralized, electron-dense, heterotopic bone usually reaches its maximum extent and density by approximately day 14 (Figure 2, C and D). Notably, the time course of mature, mineralized heterotopic bone formation is substantially prolonged in these JAB0505-treated FOP mice (Figure 2, C and D). This is evident at day 14 after injury, when most of the radiographically detectable tissue is below the threshold set for mineralized bone (Figure 2D). Indeed, the overt heterotopic bone is often scattered or lacy in appearance by μCT imaging and comprises only a small fraction of the lesional area at this time (Figure 2D). By 21 days after injury, mineralized lesions had grown substantially in JAB0505-treated mice, with an average bone volume approximately 20-fold greater than untreated FOP mice (Figure 2C). Remarkably, bony lesions of JAB0505-treated mice continued to grow up through the experimental endpoint of 35 days after injury (Figure 2, C and D, and Supplemental Figure 4), although quantification was impractical at this endpoint due to the intimate association of heterotopic bone with the limb skeleton. These data suggest that the mechanisms that normally render heterotopic bone growth self-limiting are inoperative or severely impaired in JAB0505-treated mice.

### A broadened domain of cell recruitment and delayed skeletal differentiation contribute to the pathology of JAB0505-treated Acvr1^tnR206H/+^; Tie2-Cre FOP mice.

**Figure 3:**
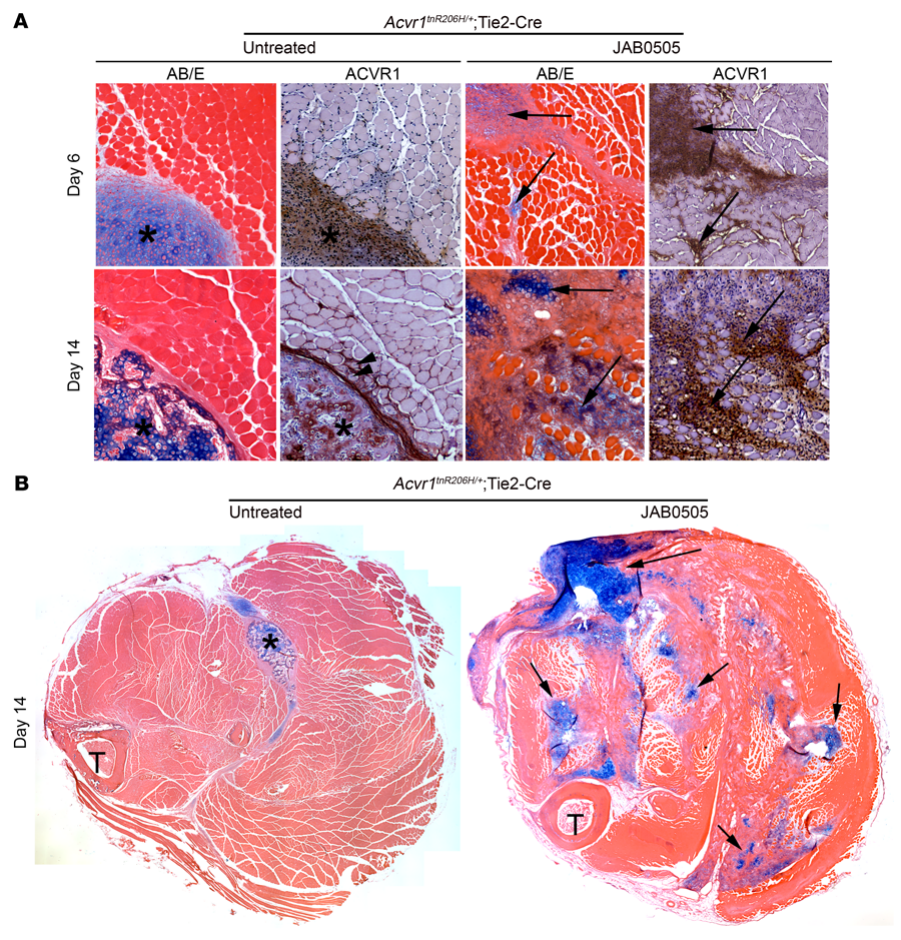
Histological comparison reveals a broadened domain of heterotopic lesion formation in JAB0505-treated FOP mice. (**A**) Transverse sections of muscle from untreated and JAB0505-treated *Acvr1^tnR206H/+^*; Tie2-Cre mice on days 6 and 14 after muscle pinch injury. Alcian blue staining to detect cartilage (blue) and immunohistochemical staining to detect ACVR1 (brown) were performed on nearby sections. Sections processed for Alcian blue were counterstained with eosin, and sections processed for ACVR1 immunohistochemistry were counterstained with hematoxylin. On day 6 after injury, untreated *Acvr1^tnR206H/+^*; Tie2-Cre mice exhibited a spatially discrete lesional region (asterisk) that was primarily comprised of ACVR1-positive ectopic cartilage. By day 14, the lesional region (asterisk) of untreated *Acvr1^tnR206H/+^*; Tie2-Cre mice displayed sporadic ACVR1 localization and was composed of both cartilage and morphologically apparent bone. In contrast, JAB0505 treated *Acvr1^tnR206H/+^*; Tie2-Cre mice displayed multiple apparent cartilaginous lesions and broader distribution of ACVR1 localization on days 6 and 14 (arrows). Centrally located myofiber nuclei (arrowheads), which identify regenerated fibers, were rare in *Acvr1^tnR206H/+^*; Tie2-Cre mice, and undetected in JAB0505-treated *Acvr1^tnR206H/+^*; Tie2-Cre mice. AB/E, Alcian blue/eosin. Original magnification, ×100. (**B**) Transverse sections of lower hind limbs of *Acvr1^tnR206H/+^*; Tie2-Cre mice 14 days after injury. Alcian blue staining revealed numerous cartilaginous lesions (blue, examples at arrows) in injured muscle of JAB0505-treated mice. Sections were counterstained with eosin. T, tibia. Original magnification, ×40.

Histological analyses revealed additional differences in response to muscle injury between JAB0505-treated and untreated *Acvr1^tnR206H/+^*; Tie2-Cre FOP mice. In contrast to the robust regenerative response of wild-type skeletal muscle (Supplemental Figure 5), muscle pinch injury of untreated FOP mice resulted in the development of histologically identifiable cartilage by day 5 or 6, and the subsequent gradual replacement of cartilage with bone beginning at approximately day 10 (Figure 3A and ref. 6). Following injury, lesional growth typically proceeds from a primary area of mesenchyme accumulation, ultimately leading to heterotopic cartilage and bone that is clearly demarcated from surrounding muscle tissue not actively engaged in skeletogenesis (6). By day 6 after injury, cartilage lesions of *Acvr1^tnR206H/+^*; Tie2-Cre mice are well defined and stain intensely with Alcian blue, which binds acidic mucopolysaccharides and glycoproteins of the cartilage matrix (Figure 3A). This contrasts with JAB0505-treated mice, in which cartilage lesions on day 6 had a less mature morphology and stained weakly with Alcian blue (Figure 3A), indicating that the formation or maturation of cartilage is delayed by JAB0505 treatment. Also apparent was a lower relative representation of mineralized bone in JAB0505-treated FOP mice on day 14 after injury. At this stage, lesions of JAB0505-treated FOP mice predominantly consisted of cartilage, in contradistinction to untreated FOP mice in which lesions at this stage were primarily composed of mineralized bone, sometimes with residual hypertrophic cartilage remaining (Figure 3, A and B, and ref. 6). Strikingly, whereas day 14 lesions of untreated FOP mice were typically composed of a contiguous bony mass, numerous Alcian blue–positive cartilage foci were scattered throughout much of the hind limb musculature of JAB0505-treated mice after a single pinch of the gastrocnemius muscle (Figure 3, A and B). Muscle integrity was broadly and severely diminished, with only scattered muscle fibers remaining in some areas (Figure 3, A and B), an effect that is probably due to the more widely disseminated pathological response of JAB0505-treated mice.

The difference in the anatomical distribution of cartilage on day 14 was reflected in the pattern of ACVR1 protein expression on day 6, when small foci of high ACVR1 expression were widely distributed in injured muscle of JAB0505-treated mice (Figure 3A). We previously showed that ACVR1 protein expression in FOP mice is upregulated shortly after injury in cells of interstitial regions that coexpress the chondrogenic marker, SOX9, and marks heterotopic skeletal tissues at later stages (6). Based on these observations, we have proposed that these connective tissue regions are areas of skeletal progenitor cell recruitment that help drive lesional growth (6). Collectively, these data suggest a model whereby treatment with JAB0505 exacerbates HO in FOP mice by expanding the anatomical domain and temporal window of FAP recruitment to skeletogenic lineages.

**Figure 4:**
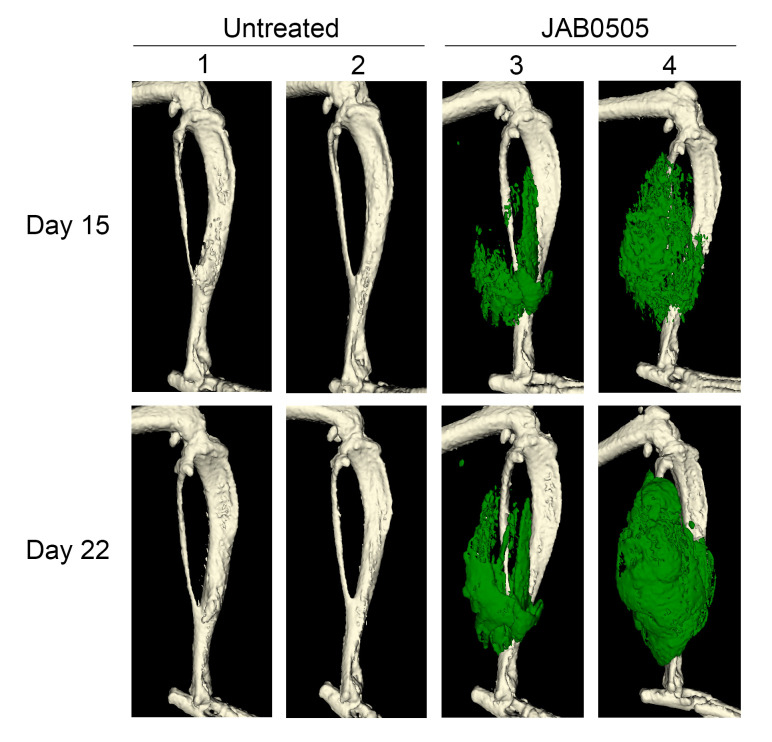
JAB0505 lowers the injury threshold necessary to cause HO in *Acvr1^tnR206H/+^*; Tie2-Cre FOP mice. μCT images of the distal hind limbs of 4 *Acvr1^tnR206H/+^*; Tie2-Cre FOP mice (numbered 1–4) at the indicated time points after injection of 50 μL of 2.5% methylcellulose into the tibialis anterior muscle, with and without administration of 10 mg/kg JAB0505 (*n =* 2 mice, 4 injected limbs, for each group). HO is pseudocolored green. A lateral view of the right hind limb of each mouse is shown. Contralateral hind limbs (not shown) received equivalent injuries and the extent of HO was comparable.

Despite its potency in exacerbating HO following injury, JAB0505 alone was not sufficient to trigger heterotopic bone formation in the absence of injury. To address whether JAB0505 could potentiate the effect of a mild, subclinical injury (i.e., insufficient to induce HO), we injected the tibialis anterior muscle of *Acvr1^tnR206H/+^*; Tie2-Cre mice with 50 μL of 2.5% methylcellulose, an injury modality that does not elicit HO in this FOP mouse model (Figure 4 and ref. 6). Remarkably, administration of JAB0505 on the day of methylcellulose injection resulted in a dramatic HO response that was similar in magnitude to its effect following muscle pinch or cardiotoxin injection (Figure 4). Evidence is presented below that JAB0505 both potentiates the inflammatory response after injury and acts as a receptor agonist, both of which likely contribute to its capacity to amplify a minor, subclinical muscle injury and induce HO.

### The effects of JAB0505 on skeletal differentiation and ACVR1 activation are ligand, cell, and context dependent.

We next explored the relative responsiveness of wild-type and R206H-FAPs to activin A and BMP6, singly and in combination with JAB0505. JAB0505 was effective at blocking BMP6-induced osteogenic differentiation of wild-type FAPs (Supplemental Figure 6A) but only partially reduced p-SMAD1/5/8 levels in BMP6-treated cells, despite molar ratios of JAB0505/ligand of 70:1 and higher (Supplemental Figure 6B). Although JAB0505 reduced activin A–driven SMAD1/5/8 phosphorylation in R206H-FAPs to a comparable degree (Supplemental Figure 6B), it did not appreciably inhibit osteogenic differentiation (Supplemental Figure 6A). The apparent disparity between effects of these ligands, and between short-term assays of receptor activation and long-term differentiation assays, suggests the engagement of multiple receptors, pathways, or both, in a context-dependent manner. In addition to ACVR1, BMP6 interacts with the SMAD1/5/8-activating receptors ALK3 and ALK6 (19), both of which are expressed by FAPs (SJ Stoessel, unpublished observations). Further, KO of ACVR1 in mouse embryonic fibroblasts inhibited chondrogenic differentiation in micromass cultures, even though p-SMAD1/5 was induced, presumably through ALK3 (10). That efficient blockade of ligand-induced ACVR1 receptor activation by JAB0505 in ACVR1(R206H)-expressing C2C12 cells (Figure 1D) does not predict the effects of JAB0505 in FAPs, highlights the complexity of BMP signaling, and demonstrates the importance of characterizing the downstream effects of receptor activation and treatment with presumptive inhibitors in cells relevant to FOP.

### JAB0505 functions as a weak agonist of ACVR1(R206H) in FAPs.

**Figure 5:**
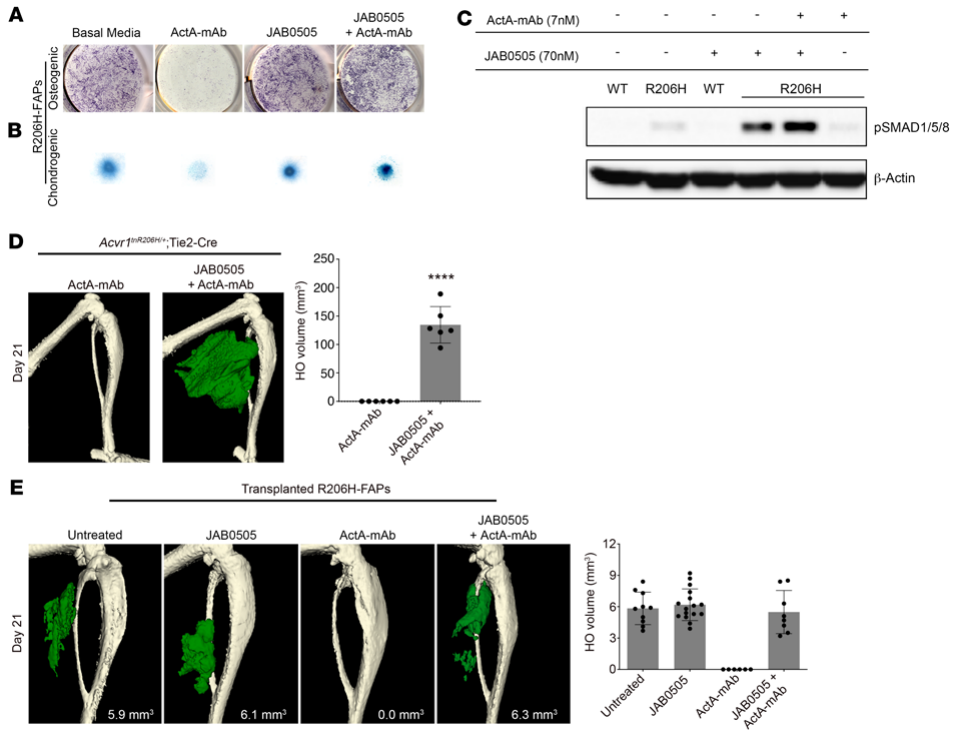
JAB0505 functions as an agonist of ACVR1(R206H). (**A**) Osteogenic differentiation of monolayer R206H-FAP cultures, as assessed by ALP staining (purple), and (**B**) chondrogenic differentiation of micromass cultures assessed by Alcian blue staining. ActA-mAb was used at 1 μg/mL (7 nM) and JAB0505 was used at 10 μg/mL (~70 nM). (**C**) Western blot of phosphorylated SMAD1/5/8 (p-SMAD1/5/8) in wild-type (WT) and R206H-FAPs (R206H). β-Actin was used as a loading control. (**D**) μCT of the distal hind limb of *Acvr1^tnR206H/+^*; Tie2-Cre mice on day 21 after injury. At the time of muscle injury, mice were treated with ActA-mAb (10 mg/kg) alone or ActA-mAb with JAB0505 (10 mg/kg). HO is pseudocolored green, and quantification is shown. ActA-mAb, *n =* 6; JAB0505 plus ActA-mAb, *n =* 6. Error bars represent ±SD. ****P < 0.0001 by 2-tailed, unpaired *t* test. (**E**) μCT images of the distal hind limb 21 days after transplantation of R206H-FAPs into the injured gastrocnemius of SCID hosts. ActA-mAb (10 mg/kg) and JAB0505 (10 mg/kg) were administered at the time of transplantation. HO is pseudocolored green and quantified, with error bars representing ±SD. Untreated, *n =* 10; JAB0505, *n =* 16; ActA-mAb, *n =* 6; JAB0505 plus ActA-mAb, *n =* 8.

As activin A is an obligatory ligand for HO formation in FOP mice (4, 6), we sought to test whether the effects of JAB0505 are dependent on activin A in culture and in vivo. As previously shown (6, 12), R206H-FAPs, but not wild-type FAPs, undergo a low level of osteogenic and chondrogenic differentiation without addition of exogenous ligand, a response that is completely blocked by an anti–activin A antibody (ActA-mAb), indicating that activin A is present in the culture media (Figure 5, A and B). Notably, JAB0505 is sufficient to drive a comparable degree of chondrogenic and osteogenic differentiation of R206H-FAPs when serum activin A is neutralized with ActA-mAb (Figure 5, A and B), demonstrating that JAB0505 functions as a weak receptor agonist in this setting. Western blot analysis of SMAD1/5/8 phosphorylation confirmed activation of BMP signaling in R206H-FAPs by JAB0505 in the absence of activin A (Figure 5C), and this was also observed when JAB0505 was used at the same concentration as activin A (1 nM; data not shown). JAB0505 did not induce skeletal differentiation or SMAD1/5/8 phosphorylation of wild-type FAPs (Figure 5C and Supplemental Figure 6A), indicating that its agonist activity is functionally similar to activin A in that it activates BMP signaling only through ACVR1(R206H). This weak agonist activity can also partially explain the inability of JAB0505 to block activin A–induced skeletogenic differentiation and SMAD1/5/8 phosphorylation in cultured R206H-FAPs (Supplemental Figure 6, A and B).

We next tested whether JAB0505 can function as a receptor agonist in FOP mice. The gastrocnemius muscle of *Acvr1^tnR206H/+^*; Tie2-Cre FOP mice was pinch injured, and mice were treated with 10 mg/kg JAB0505 at the time of injury, with or without 10 mg/kg ActA-mAb, a dose that blocks injury-induced HO in this model (6). FOP mice treated with both JAB0505 and ActA-mAb also developed explosive HO (Figure 5D), indicating that JAB0505 can replace the essential functions of activin A in driving the formation and growth of injury-triggered HO. Further, these results suggest that neoactivation of ACVR1(R206H) is the only obligate role of activin A in driving HO in FOP, and that activin A’s native p-Smad2/3–mediated signaling function, which can induce chondrogenic differentiation of embryonic preskeletal progenitor cells (20), is dispensable in this context.

As cells in addition to FAPs are recombined in *Acvr1^tnR206H/+^*; Tie2-Cre mice, including endothelial cells and CD45^+^ hematopoietic cells (6, 17, 18, 21), we further assessed FAPs as a target of receptor agonist properties of JAB0505 in transplantation assays. FAPs were isolated, expanded in culture, and transplanted into the preinjured gastrocnemius muscle of SCID mice, as previously described (6, 12). Transplanted R206H-FAPs consistently formed heterotopic bone, as assayed by μCT on day 21 after transplantation (Figure 5E), and injection of ActA-mAb on the day of transplantation completely blocked osteogenic differentiation (Figure 5E), consistent with previous studies (6, 12). Importantly, administration of JAB0505 to ActA-mAb–treated hosts restored osteogenic differentiation of transplanted R206H-FAPs (Figure 5E), indicating that JAB0505 acts directly on R206H-FAPs and can replace the essential function of activin A in driving their skeletogenic differentiation. Production of peak heterotopic bone was modestly delayed when hosts were treated with JAB0505 (Supplemental Figure 7; day 14 vs. day 10), a result that likely reflects both the receptor-blocking and weak receptor agonist activity of JAB0505, as in FOP mice. Notably, however, JAB0505 did not increase the peak quantity of bone generated by transplanted R206H-FAPs (Figure 5E and Supplemental Figure 7), suggesting that JAB0505 does not act directly on R206H-FAPs to increase their capacity for proliferation or differentiation.

### JAB0505 treatment causes abnormal dynamics and extent of R206H-FAP expansion after muscle injury in FOP mice.

**Figure 6:**
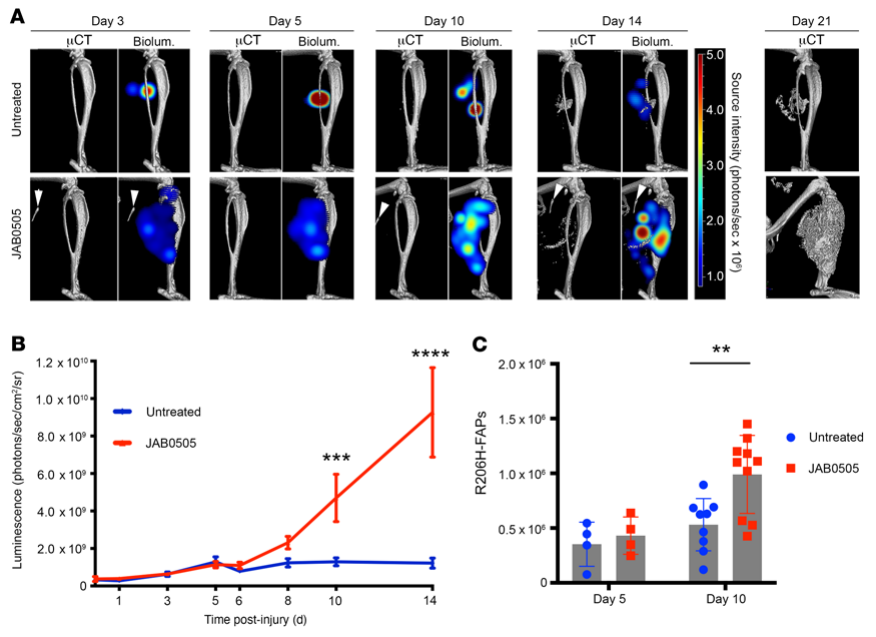
JAB0505 treatment causes a sustained increase in R206H-FAPs following skeletal muscle injury of FOP mice. (**A**) 3D tomographic bioluminescence source reconstruction following muscle pinch injury of *Acvr1^tnR206H/+^*; *R26^Luc/+^*; Tie2-Cre FOP mice, with and without administration of JAB0505. Paired images show μCT alone (left panel) and μCT combined with the corresponding 3D bioluminescence reconstruction (right panel). The same mouse is shown from days 3 to 21. Bioluminescence reconstruction was not performed on day 21 due to the dampening effect of dense bone on luminescent output. (**B**) Graphical representation of bioluminescent population dynamics of Tie2^+^ cells from *Acvr1^tnR206H/+^*; *R26^Luc/+^*; Tie2-Cre mice following pinch injury. Untreated, *n =* 16; JAB0505, *n =* 10. Error bars represent ±SEM. ***P ≤ 0.001, ****P ≤ 0.0001 by 2-way ANOVA with Sidak’s multiple-comparison test. (**C**) Flow cytometry analysis to determine R206H-FAP cell number in injured distal hind limb muscles of *Acvr1^tnR206H/+^*; *R26^NG/+^*; Tie2-Cre mice that were either untreated (day 5, *n =* 4; day 10, *n =* 9) or administered JAB0505 at 10 mg/kg (day 5, *n =* 4; day 10, *n =* 10). Error bars represent ±SD. **P ≤ 0.01 by 2-tailed, unpaired *t* test with Welch’s correction.

To determine whether JAB0505 treatment causes alterations in R206H-FAP population growth dynamics in FOP mice, longitudinal live-animal luminescence imaging and flow cytometry were performed following muscle pinch injury. The Tie2-Cre driver was used to recombine the *Acvr1^tnR206H^* allele and the Cre-dependent luciferase reporter, *R26^luc^* (22). As Tie2-Cre is also expressed in hematopoietic cells and endothelium, these initial experiments provided a combined luminescent readout of 3 critical cellular events following injury: immune cell infiltration and population growth of both FAPs and endothelial cells. After days 5 or 6, R206H-FAP–derived cartilage and bone also contribute to the luminescent signal, given the permanence of the Cre/loxP labeling system. The luminescent signal increased during the first few days following injury, and treatment with JAB0505 did not have a marked effect on apparent numbers of *R26^luc^*-recombined cells at these early stages (Figure 6, A and B). However, whereas the apparent number of recombined cells in untreated FOP mice increased until day 5 and remained relatively constant thereafter, the luminescent signal from JAB0505-treated FOP mice continued to increase through 14 days after injury (Figure 6, A and B). Quantification was not undertaken on day 21 because of the dampening of luminescence output by mineralized bone, particularly in JAB0505-treated mice. Interestingly, the luminescent signal in JAB0505-treated mice extended throughout most of the injured muscle, even in the first few days after injury (Figure 6A), supporting the conclusion from histological analyses that a consequence of JAB0505 treatment is to engage muscle tissue over a much broader anatomical domain than in untreated FOP mice.

As R206H-FAP–derived cartilage and bone contribute to the luminescent signal after the onset of skeletogenic differentiation, and because Tie2-Cre does not exclusively label FAPs, flow cytometry was employed to directly quantify R206H-FAP cell numbers. R206H-FAPs were directly isolated from pinch-injured hind limb muscles of *Acvr1^tnR206H/+^*; Tie2-Cre; *R26^NG/+^* mice as CD45^–^CD31^–^SCA1^+^GFP^+^tdTomato^–^ cells (Supplemental Figure 8; see Methods), where *R26^NG^* is a Cre-dependent GFP reporter (23) and tdTomato identifies cells unrecombined at the *Acvr1^tnR206H^* locus (6). R206H-FAP numbers were similar between untreated and JAB0505-treated FOP mice at 5 days after injury (Figure 6C), the approximate onset of cartilage differentiation. On day 10, when skeletogenic differentiation is advanced, the average number of R206H-FAPs was almost 2-fold greater in JAB0505-treated mice (Figure 6C). As HO volume at endpoint in transplantation assays is directly correlated with peak R206H-FAP numbers (12), these data support the notion that JAB0505 exacerbates HO, in part, by acting to increase the number of R206H-FAPs that ultimately undergo skeletogenic differentiation. Although the increase in FAPs was not proportional to the profound increase in HO at endpoint, this may be explained by a sustained dynamic equilibrium between R206H-FAP population growth driven by recruitment, proliferation, and reduced apoptosis (24), and loss of these skeletal progenitors as they underwent commitment and differentiation to cartilage and bone. Additionally, the calculated increase in R206H-FAP numbers on day 10 likely underestimated the increase in HO-forming regions, as quantification was conducted using total musculature of the posterior hind limb given the difficulty of identifying presumptive HO-forming regions with precision.

### Dysregulation of the immunological response to muscle injury in JAB0505-treated FOP mice.

**Figure 7:**
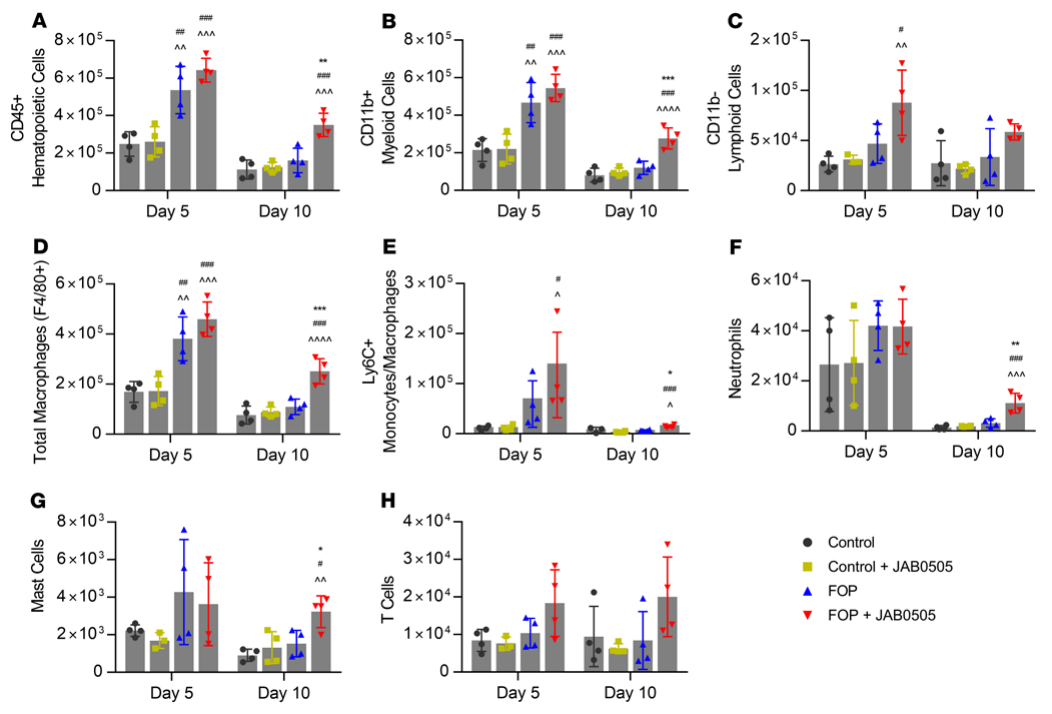
JAB0505 treatment causes a sustained increase in several immune cell populations following skeletal muscle injury of *Acvr1^tnR206H/+^*; Tie2-Cre FOP mice. Flow cytometry analysis was used to determine cell numbers of (**A**) total CD45^+^ hematopoietic cells, (**B**) myeloid cells, (**C**) lymphoid cells, (**D**) total macrophages, (**E**) Ly6C^+^ inflammatory monocytes/macrophages, (**F**) neutrophils, (**G**) mast cells, and (**H**) T cells in injured distal hind limb muscles of control (*R26^NG/+^*; Tie2-Cre) and FOP (*Acvr1^tnR206H/+^*; *R26^NG/+^*; Tie2-Cre) mice that were either untreated or administered 10 mg/kg JAB0505 i.p. (*n =* 3–4). Error bars represent ±SD. Significance was determined using 1-way ANOVA with Tukey’s multiple-comparison test within individual time points. Symbols representing significance were placed above FOP and FOP + JAB0505 bars to indicate a comparison to control (^), control + JAB0505 (^#^), and FOP (*). The numbers of symbols of each type denote levels of significance: (1) *P ≤* 0.05, (2) *P ≤* 0.01, (3) *P ≤* 0.001, and (4) *P ≤* 0.0001. No control vs. control + JAB0505 comparisons were significant.

Given the well-accepted association between inflammation and HO flare-ups in FOP patients (25, 26), we assessed the effect of JAB0505 on several immune cell populations previously implicated in HO (27) and FOP pathogenesis (26). Single cells were isolated from total posterior hind limb skeletal muscle of JAB0505-treated and untreated FOP (*Acvr1^tnR206H/+^*; Tie2-Cre; *R26^NG/+^*) and control (Tie2-Cre; *R26^NG/+^*) mice on days 5 and 10 after pinch injury. Within the encompassing CD45^+^ hematopoietic population, we quantified myeloid cells (CD11b^+^), lymphoid cells (CD11b^–^), total macrophages (CD11b^+^Ly6G^–^F4/80^+^), inflammatory monocytes/macrophages (CD11b^+^Ly6G^–^F4/80^+/–^Ly6C^+^), neutrophils (CD11b^+^Ly6G^+^), mast cells (CD11b^–^FcεR1α^+^CD117^+^), and T cells (CD11b^–^CD3^+^) (Supplemental Figure 9). JAB0505 treatment did not significantly alter representation of any of the assessed immune populations in uninjured mice (Supplemental Figure 10) or in non-FOP littermates (controls) on either day 5 or 10 after injury (Figure 7, A–H).

Compared with littermate controls at 5 days after injury, the number of CD45^+^ hematopoietic, total myeloid, and total macrophage populations in untreated FOP muscle was significantly elevated (Figure 7, A, B, and D), and the majority of the remaining populations trended higher (Figure 7, A–H). Supporting this observation, immunohistochemical analysis of lesional tissue also documented elevation of several immune cell populations in FOP mice at a similar time after injury (26). Following treatment of FOP mice with JAB0505, most immune cell populations trended higher, but were not significantly different than untreated FOP mice 5 days after injury (Figure 7, A–H). By 10 days after injury, cell numbers of all immune populations in untreated FOP muscle returned to control levels (Figure 7, A–H). Importantly, however, most immune cell populations remained significantly elevated in JAB0505-treated FOP muscle, with neutrophils approximately 3.5-fold higher and CD45^+^ hematopoietic, total myeloid, total macrophage, Ly6C^+^ inflammatory monocytes/macrophage, and mast cell populations each elevated over 2-fold compared with injured muscle of untreated FOP mice (Figure 7, A–H). Collectively, these data demonstrate that treatment of FOP mice with JAB0505 causes a sustained, injury-dependent immunological reaction of a duration that extends beyond the dysregulated response observed in untreated FOP mice.

## Discussion

The present study demonstrated that an antibody raised against the ECD of ACVR1 has the unexpected property of profoundly exacerbating HO in 2 mouse genetic models of FOP. We showed that while JAB0505 effectively inhibits ligand-dependent BMP signaling through wild-type ACVR1 and ACVR1(R206H) in certain cell contexts in vitro, JAB0505 also functions as a weak agonist of ACVR1(R206H), thereby replacing the essential function of activin A in injury-induced skeletal lesion formation in FOP mice (4, 6). Recent independent studies confirmed the ability of anti-ACVR1 antibodies to profoundly worsen HO in FOP mice, and further demonstrated that the HO-exacerbating effects were dependent on antibody bivalency and their ability to cluster ACVR1(R206H)-containing receptor complexes (14). Collectively, these studies raise serious safety and efficacy concerns for the use of anti-ACVR1 antibodies as a treatment modality to block ligand-receptor interactions in patients with FOP.

Exacerbation of HO was observed both when the conditional *Acvr1^R206H^* allele was expressed in its normal expression pattern, which includes a broad spectrum of cell types (28), as well as when Tie2-Cre–dependent recombination narrowed the known range of cell types capable of expressing the FOP allele to FAPs, endothelial cells, and hematopoietic cells (17, 18). Among these latter cell types, only FAPs contribute to skeletal lesions in FOP mouse models (6, 13, 18), or in response to supraphysiological levels of BMP2 (18, 21, 29). Thus, although we cannot formally rule out the contribution to HO of other Tie2-expressing cell types in JAB0505-treated mice, available evidence supports the conclusion that FAPs are primarily responsible for the exacerbated HO phenotype in this model. HO lesions in *Acvr1^tnR206H/+^*; Tie2-Cre mice are composed almost exclusively of mutant cells (recombined at the *Acvr1^tnR206H^* locus), despite the preponderance of unrecombined FAPs in native and injured muscle tissue (~80%–90%; ref. 6) due to inefficient Tie2-Cre–driven recombination of this locus in FAPs (6). This cell-autonomous requirement for ACVR1(R206H), together with the finding that JAB0505 functions as an agonist of ACVR1(R206H), but not wild-type ACVR1, specifically implicates ACVR1(R206H)-expressing FAPs as the primary cellular target of JAB0505 action in these FOP mice.

We suggest a model whereby treatment with JAB0505 exacerbates HO in FOP mice by expanding the anatomical domain and temporal window of R206H-FAP activation, proliferation, and recruitment to skeletogenic lineages. This model is supported by histological analyses of JAB0505-treated *Acvr1^tnR206H/+^*; Tie2-Cre FOP mice, which showed multiple foci of lesional tissue across most of the lower hind limb following a single pinch injury, by the significantly elevated number of R206H-FAPs present on day 10 after injury, and by the prolonged period of lesional growth that typifies the HO response in these mice. In this latter regard, while the timing of the initial appearance of cartilage and mineralized bone was similar in treated and untreated mice, the relative fraction of lesional tissue composed of fibroproliferative cells, cartilage, and unmineralized bone was greater and persisted for a much longer period after injury in JAB0505-treated FOP mice. These population-level observations are a predicted consequence of prolonged R206H-FAP recruitment and proliferation, which would provide an ongoing source of new cartilage and bone progenitors over time.

The finding that JAB0505 treatment of SCID hosts did not potentiate HO following transplantation of R206H-FAPs also supports this model. Muscle-resident progenitors of transplantation hosts should lack the capacity to contribute to HO because *Acvr1^R206H^* functions largely cell autonomously (6), and JAB0505′s agonist activity is restricted to ACVR1(R206H)-expressing cells. That JAB0505 did not worsen HO when a defined number of responsive cells was placed in a “non-recruitable” environment provides further, albeit indirect support for the relevance of enhanced and sustained progenitor cell recruitment as a mechanism of JAB0505 action following muscle injury in FOP mice. A priori, a direct positive effector function of JAB0505 in the proliferation of R206H-FAPs or their derivatives is a non–mutually exclusive alternative model that could explain the exacerbating effects of JAB0505 in FOP mice. However, the inability of JAB0505 to enhance HO in this transplantation model, where HO volume at endpoint is directly correlated with peak R206H-FAP number (12), argues against a primary role of JAB0505 in stimulating or sustaining proliferation of R206H-FAPs or their skeletal progenitor descendants.

The finding that JAB0505 functions as an agonist of ACVR1(R206H) raises an apparent paradox. Whereas the receptor-activating capacity of JAB0505 is demonstrably weaker than that of activin A in cell culture assays, JAB0505 nevertheless profoundly worsened HO relative to the activin A–driven process in untreated FOP mice. We propose that the tissue distribution and bioavailability of JAB0505 is at least partly responsible. In fact, the observations of broader engagement of muscle-resident skeletal progenitors, delayed formation of heterotopic bone, and a prolonged period of HO growth are consistent with broad spatial distribution of a weakly activating but long-acting agonist antibody. By extension, these considerations would predict that the more restricted HO response in FOP mice reflects the spatiotemporal restriction of endogenous activin A, localized sources of which may include macrophages, other immune cells, and fibroblasts that accumulate at the site of injury (20, 30).

Further insights into JAB0505′s actions came from the capacity of JAB0505 to cause explosive HO following intramuscular injection of methylcellulose, which otherwise does not elicit an HO response (6). Specifically, this capacity to amplify a subclinical injury suggests the general model that JAB0505 reduces the injury threshold required to fully engage R206H-FAPs (and perhaps other progenitors) in endochondral bone formation. This function would predict more widely disseminated disease by recruitment of skeletal progenitors at a greater distance from a focal muscle pinch injury. As JAB0505 does not cause HO in the absence of injury, it is reasonable to assume that R206H-FAPs experiencing even a subthreshold injury (methylcellulose-treated limb or cells distant from the site of pinch injury) enter a fully responsive developmental state such that they are poised for direct recruitment into the endochondral pathway upon further stimulation by JAB0505. Alternatively, these R206H-FAPs may enter a G_alert_-like state (31) such that responsiveness to JAB0505 and entry into the endochondral pathway requires an additional stimulus. G_alert_ was originally described by Rando and colleagues as a transitional and normally adaptive cell state induced by systemic injury factors that positions stem cells at distant anatomical regions (e.g., in the limb contralateral to the site of injury) to respond rapidly to subsequent injury and stress (31). Given that alterations in the inflammatory environment can activate the osteogenic transcriptional program of FAPs in a model of acquired HO (29), and that tissue inflammation is a well-known trigger for HO in FOP (25, 26), one possibility is that an enhanced or altered immune response after muscle injury constitutes the additional needed stimulus. Thus, in addition to its agonist properties, JAB0505 directly or indirectly amplifies and sustains the local inflammatory response caused by muscle injury. The localized nature of such an inflammatory response would explain why systemically distributed JAB0505 only causes HO in the injured limb of *Acvr1^tnR206H/+^*; Tie2-Cre FOP mice, despite the presence of R206H-FAPs in musculature throughout the body. Immune system involvement is consistent with the significant and sustained elevation of several hematopoietic cell populations in muscle tissue 10 days after injury in JAB0505-treated mice, a time point at which these immune cell populations had returned to control levels in untreated FOP mice. Notably, combined depletion of macrophages and mast cells substantially reduced injury-induced HO in an independent FOP mouse model (26), potentially implicating their dysregulated response to muscle injury in the exacerbating effects of JAB0505 treatment. In addition, dysregulation of the inflammatory response may also explain the increased number of R206H-FAPs in JAB0505-treated mice, as immune system–imposed mechanisms normally regulate FAP numbers after injury (32, 33). Although immune-modulatory activities of JAB0505 remain to be defined, interactions with immune cells through the Fc region of JAB0505 (or other anti-ACVR1 antibodies) is one possible mechanism of action. Clearly, however, JAB0505 treatment alone is not sufficient to alter the immunological response to muscle injury, as immune cell populations were unaffected in control mice treated with JAB0505.

Understanding the pathological effects of anti-ACVR1 antibodies will require follow-up investigations that explore the connection between antibody-mediated receptor clustering (14), downstream biochemical sequelae, and the tissue-level cellular effects reported here. Regardless of mechanism, these studies demonstrate that anti-ACVR1 antibodies characterized to date profoundly exacerbate disease progression in FOP mice, highlighting the importance of using genetically and physiologically relevant mouse models and cell types to evaluate potential therapeutic candidates for FOP.

## Methods

### Ligands and ActA-mAb

Activin A (338-AC), BMP2 (355-BM-010), mouse BMP4 (5020-BP-010), recombinant human (rh) BMP4 (314-BP/CF), mouse BMP6 (6325-BM [Supplemental Figure 2] or 507BP [Supplemental Figure 6]) mouse BMP7 (5666-BP-010), mouse BMP9 (5566-BP-010), rhBMP9 (3209-BP/CF), and mouse BMP10 (6038-BP-025) were purchased from R & D Systems. In the methods below, ligands were of mouse origin unless stated otherwise. ActA-mAb was provided by Acceleron Pharma and was described previously (6).

### Discovery and optimization of the anti-ACVR1 mAb

The anti-ACVR1 mAb was obtained from a phage display library of Fab fragments cloned from mice immunized with DNA encoding the ACVR1 ECD. CD-1 and NZBWF/J mice were immunized by subcutaneous injection of ACVR1 ECD plasmid DNA suspended in sterile TE buffer supplemented with aSMART DNA immunization adjuvant reagent (Antibody Research, 113010) or with ACVR1 ECD plasmid DNA that was precipitated onto gold beads and administered using a HELIOS Gene Gun (Bio-Rad).

Anti-ACVR1 specific sera titers from immunized mice were determined by ELISA. ELISA plates were coated with a mixture of recombinant human ACVR1 antigens consisting of the ACVR1 ECD fused to human G1 Fc (Sino Biologicals, 10227-H03B), the human ACVR1 ECD fused to human G1 Fc (R&D Systems, 637-AR), and the ACVR1 ECD (Creative Biomart, ACVR1-01H). Antibodies bound to ACVR1 were detected using goat anti–mouse IgG (H+L), HRP (Thermo Fisher Scientific, 62-6520).

A Fab phage display library was constructed from ACVR1-immunized, sera-positive mice. Total RNA was isolated from lymph nodes and spleens. This was used as the template for cDNA synthesis using Superscript III First-Strand Synthesis System for RT-PCR (Life Technologies). The cDNA was used as the template for the amplification of heavy chain IgG1, IgG2a, and light chain Ig κ sequences. The antibody sequences were cloned in bulk into a phage display vector. The phagemid was used to transform XL1-blue *E*. *coli* cells to generate the anti-ACVR1 library.

The library was panned for 2 rounds on biotinylated ACVR1 ECD (Creative Biomart) that was conjugated to magnetic streptavidin beads (Dynabeads MyOne, Thermo Fisher Scientific, 65601). Streptavidin beads without ACVR1 were used in deselection steps to remove nonspecific binders. Phage eluted from ACVR1-coated beads were tested for binding to the ACVR1 ECD using an electrochemiluminescence immunoassay (Meso Scale Discovery). The variable region gene sequences of phage that bound to ACVR1 were cloned into mammalian expression plasmids for the expression of full-length mouse IgG1 κ mAbs.

Full-length antibodies were expressed in HEK293 cells and purified by protein A chromatography. Antibodies were tested for binding to the ACVR1 ECD by biolayer interferometry using an Octet HTX instrument (Pall/Forte Bio). Confirmed binders were evaluated for the ability to block BMP-mediated ACVR1 signaling in a cell-based reporter assay described below.

Affinity maturation of functional anti-ACVR1 antibodies was performed by mutating the CDRs by saturation mutagenesis of each position of heavy chain CDR2 and -3 and light chain CDR3 independently using primers that randomly encode NNK at each specified codon, where *N =* A/C/G/T and K = G/T. Mutated antibody sequences were pooled and cloned into a phagemid vector to generate an affinity maturation library. This phage library was panned for ACVR1 binders for 4 rounds of increasingly stringent binding conditions. Phage binders were sequenced, and convergent antibody sequences were cloned and expressed as murine IgG1 mAbs. Antibodies were expressed and screened for binding to ACVR1 by BIAcore and evaluated for inhibition of BMP2-, 4-, 6-, 7-, 9-, and -10-mediated ACVR1 signaling using the BMP response element (BRE) luciferase reporter assays described below. All BMPs were tested in reporter cells expressing wild-type ACVR1 alone. In addition, inhibition of BMP9-mediated signaling was determined in reporter cells that express both wild-type ACVR1 and ACVR1(R206H). The specificity of affinity-matured antibodies for ACVR1 was confirmed by flow cytometry using wild-type ACVR1–expressing and ACVR1-KO C2C12 cells.

The amino acid sequence of antibody JAB0505 has been published in US Patent Application, “Anti-ALK2 Antibodies and Uses Thereof,” patent number PCT/US 20 19/064613, WO 2020/118011 A1, and is shown in Supplemental Figure 1.

### Wild-type C2C12 BMP-reporter cell line development

A BMP reporter plasmid was constructed by synthesizing a BRE described previously (15). The BRE was inserted upstream of the minimal promotor region of plasmid pGL4.26 luc2/minP/Hygro (Promega, E8441) that also encodes the luciferase reporter gene *luc2*. The sequence of the BRE was confirmed by DNA sequencing. The plasmid was designated pGL4.26 BRE2. Vector construction and sequence confirmation were performed by GeneArt (Thermo Fisher Scientific).

Mouse C2C12 cells (ATCC, CRL-1772) were transfected with plasmid pGL4.26 BRE2 using Lipofectamine 3000 (Thermo Fisher Scientific, L300015) according to the manufacturer’s instructions. Transfected cells were cultured in 96-well plates in DMEM with high glucose and L-glutamine (ATCC, 30-2002), 10% FBS (Tissue Culture Biology 101), and 200 μg/mL hygromycin (Thermo Fisher Scientific, 10687-010). Stable clones were scaled up and cryopreserved.

Transfected C2C12 clones were evaluated for BMP-dependent luciferase expression by plating cells at 2.0 × 10^4^ viable cells per well in 96-well plates in selective media containing 1% FBS. Cells were stimulated with titrations of mouse BMP9 ranging from 10 ng/mL to 156 pg/mL. Luciferase expression was analyzed using the Dual-Glo Luciferase assay system (Promega, E2940). Luminescence was read with either a SpectraMax Paradigm or Flexstation 3 plate reader (both Molecular Devices). Clone 21E12 was found to produce the highest luminescence in response to BMP9. It was also found to stably respond to BMP9 after 41 generations and was selected for use in cell-based assays measuring inhibition of wild-type ACVR1. Clone 21E12 was also evaluated for BMP-dependent luciferase expression upon stimulation with titrations of mouse BMP2, -4, -6, -7, and -10 ranging from 80 ng/mL to 37 pg/mL.

### Development of ACVR1(R206H)-transfected C2C12-BRE-Luc reporter cell line

A synthetic DNA construct encoding mouse ACVR1 with the R206H mutation (G to A transition at nucleotide position 617) was assembled from synthetic oligonucleotides and PCR products and cloned into the pCMV6 entry mammalian expression vector (Origene), which also encodes a neomycin-resistance marker. This vector was designated pCMV6-ALK2R206H. Vector construction and sequence verification were performed by GeneArt (Thermo Fisher Scientific).

C2C12 cells were transfected with plasmid pCMV6-ALK2R206H using Lipofectamine 3000 according to the manufacturer’s instructions. Transfected cells were cultured in 96-well plates in selective media containing 400 μg/mL geneticin (Life Technologies, 10131-027). Stable clones were scaled up in selective media and cryopreserved.

Clones were analyzed for increased cell surface expression of ACVR1 relative to wild-type C2C12 cells by flow cytometry. Cells were washed once in sterile PBS and stained with polyclonal anti–human activin A receptor type 1 antibody (LSBio, LS-C122594) conjugated to Zenon Alexa Fluor 647 (Molecular Probes, Z25608). Labeled cells were analyzed by flow cytometry using an LSRII flow cytometer (BD Biosciences). Transfected clones that had a higher ACVR1 mean fluorescence intensity (MFI) than nontransfected C2C12 cells were transiently transfected with the BRE reporter plasmid pGL4.26 BRE2 described above using Lipofectamine LTX (Life Technologies, 15338-100) according to the manufacturer’s instructions. Transfected cells were cultured overnight in media containing 1% FBS in 96-well plates. The following day, clones were treated with a dilution series of recombinant mouse BMP9 ranging from 100 ng/mL to 0.1 ng/mL (4.1 nM to 4.1 pM) for 4 hours. Expression of luciferase was then determined using the Dual-Glo Luciferase Assay System (Promega, E2940). Luminescence was measured with a SpectraMax Paradigm plate reader (Molecular Devices).

ACVR1(R206H)-transfected clones expressing luciferase constitutively, and at higher levels than wild-type C2C12 cells when stimulated with BMP9, were stably transfected with the BRE reporter plasmid using Lipofectamine 3000 according to the manufacturer’s instructions. Transfected cells were cultured in 96-well plates in media containing 200 μg/mL hygromycin and 400 μg/mL geneticin and cryopreserved.

Clones resistant to both hygromycin and geneticin were stimulated with BMP9 or BMP4 in media containing 1% FBS and tested for expression of luciferase using the ONE-Glo + Tox Luciferase Reporter and Cell Viability Assay (Promega, E7120). Luciferase expression was measured using a Flexstation 3 plate reader (Molecular Devices). Clone 84C7 was found to have elevated basal constitutive BRE-Luc expression and enhanced BMP9 and BMP4 BRE-Luc expression compared with the non–ACVR1(R206H)-transfected C2C12 BRE-Luc clone 21E12. Clone 84C7 was selected for use in cell-based assays measuring inhibition of ACVR1(R206H).

### Generation of Acvr1 and Bmpr1a (Alk3) KOs in C2C12 BRE reporter lines

The C2C12 BRE reporter line 21E12 was used to generate *Acvr1* (*Alk2*)- and *Bmpr1a* (*Alk3*)-KO BRE reporter lines. To generate *Acvr1*-KO and *Bmpr1a*-KO clones, 21E12 cells were cotransfected with 3 plasmids; one encoding the Cas9 enzyme, one encoding a single guide RNA (sgRNA) specific for either the ACVR1 gene (targeting exon 6 sgRNA 5′-TGTAAGACCCCGCCGTCACCTGG-3′) or the *Bmpr1a* gene (targeting exon 5 sgRNA 5′-CATTATAGAAGAAGATGATCAGG-3′), and a reporter plasmid encoding GFP and H2KK sequences that require *Acvr1* sgRNA– or *Bmpr1a* sgRNA–mediated frame shift for expression of GFP and H2KK, respectively. All plasmids were obtained from PNA Bio Inc. Cells were transfected using Amaxa 4D-Nucleofector with X-unit (Lonza). Two days after transfection, cells were enriched magnetically by their expression of H2Kk using a MACSelect Transfected Cell Selection kit (Miltenyi Biotec). Cas9-mediated indel formation at the *Acvr1* or *Bmpr1a* locus was confirmed by T7E1 mismatch analysis on the enriched cell populations (T7E1 Nuclease, New England Biolabs, M0302S). H2Kk^+^ cells were cloned by limiting dilution in 96-well plates. Clones were screened for ACVR1 or ALK3 protein expression by flow cytometry with an anti-ACVR1 antibody (R&D Systems, AF637) and an anti-ALK3 antibody (Sino Biological, 50078), respectively. *Acvr1*-KO and *Alk3*-KO clones were confirmed by genomic sequencing and by loss of response to rhBMP9 or rhBMP4, respectively.

### Characterization of BMP receptor expression in C2C12 cells

BMP receptor mRNA expression was profiled in C2C12 cells by RT-qPCR. C2C12 cells were cultured in DMEM with high glucose, L-glutamine (ATCC, 30-2002), and 10% FBS (Tissue Culture Biology 101). Total RNA was isolated from C2C12 cells using a RNeasy Plus Universal kit (Qiagen, 73404) and cDNA was synthesized using an iScript cDNA synthesis kit (Bio-Rad, 170-8890). The expression of *Acvrl1* (*Alk1*), *Acvr1* (*Alk2*), *Bmpr1a* (*Alk3*), *Bmpr1b* (*Alk6*), *Gapdh*, and *Actb* (β-actin) was quantified in triplicate by RT-qPCR using TaqMan primer and probes (Thermo Fisher Scientific). Average expression levels of *Alk1* and *Acvr1* are reported relative to *Actb* and average expression levels of *Alk3* and *Alk6* are reported relative to *Gapdh*. Expression values are presented as ΔCT, which is the difference in threshold cycle time between the housekeeping gene and the BMP type 1 receptor. Experimental variability was expressed as the standard deviation among the 3 amplification replicates.

The cell surface expression of ACVR1 and ALK3 proteins on C2C12 cells was evaluated by flow cytometry. Cells were removed from adherent cultures using AssayComplete cell dissociation reagent (Eurofins, 92-0009) and incubated with a polyclonal anti-ALK2 antibody (R&D Systems, AF637) or a polyclonal anti-ALK3 antibody (Sino Biological, 50078-RP02). The isotype control cells were ChromPure goat IgG (Jackson ImmunoResearch, 005-000-003) for anti-ALK2 and ChromPure rabbit IgG (Jackson ImmunoResearch, 011-000-003) for anti-ALK3. Cells were washed and incubated with the secondary antibodies donkey anti–goat IgG, Alexa Fluor 488 (Thermo Fisher Scientific, A-11055) and goat anti–rabbit IgG Alexa Fluor 488 (Thermo Fisher Scientific, A-11070) for anti-ALK2 and anti-ALK3, respectively. Cells were washed again and analyzed with an LSR II flow cytometer (BD Biosciences).

### FOP mouse models and crosses to generate experimental mice

#### Acvr1^FLEx(R206H)^ FOP mouse model and tamoxifen induction.

The *Acvr1^FLEx(R206H)^* tamoxifen-inducible FOP mouse model was developed by genOway. The FLEx system (34) is a Cre-dependent 1-way genetic switch that allows for expression of a mutant form of a gene in place of its wild-type copy. Briefly, *Acvr1^FLEx(R206H)^* was designed such that mutant exon 5, which carries the 617G→A substitution and was inserted in reverse orientation in intron 5, is switched to sense orientation and replaces the wild-type exon by the action of Cre. The mutant gene is expressed under the control of the endogenous *Acvr1* promoter. Electroporation of the targeting vector into 129Sv embryonic stem (ES) cells, G418 selection, and validation of targeted clones followed routine methods. Prior to production of chimeric mice by blastocyst injection, validated ES clones were transfected with a plasmid encoding Flp recombinase to remove the Frt-flanked neo cassette present in the targeting vector. Two highly chimeric males were mated with wild-type 129Sv females to establish *Acvr1^FLEx(R206H)^* mouse lines. Mice were maintained as heterozygotes on a 129Sv background.

To produce experimental mice, heterozygous *Acvr1^FLEx(R206H)^* female mice were crossed with heterozygous B6.Cg-Tg(CAG-cre/Esr1*)5Amc/J (The Jackson Laboratory, 004682) males to generate mice that were heterozygous at both loci, referred to as *Acvr1^FLEx(R206H)/+^*; CAG-Cre^ERT2^. The Cre driver CAG-cre/Esr1 was selected to provide high levels of Cre-recombinase expression across most cell types, and its activity is tamoxifen dependent. Mice between 4 and 8 weeks of age were used, and males and females were randomly assigned to study groups.

To induce recombination at the *Acvr1^FLEx(R206H)^* locus, 75 mg/kg tamoxifen (Sigma-Aldrich, T5648) prepared as a 20 mg/mL stock in corn oil (Sigma-Aldrich, C8267) was administered to experimental mice for 5 consecutive days. A 5- to 7-day washout period was incorporated prior to muscle injury.

#### Acvr1^tnR206H^ FOP mouse model.

Generation of *Acvr1^tnR206H^* mice was previously described (6), and standard breeding schemes were used to produce mice that carry the Tie2-Cre driver (17) [B6.Cg-Tg(Tek-cre)1Ywa/J; The Jackson Laboratory, 008863] and the reporters, *R26^NG^* (23) [derived from *R26^NZG^*; FVB.Cg-*Gt(ROSA)26Sor^tm1(CAG-lacZ,-EGFP)Glh^*/J; The Jackson Laboratory, 012429] or *R26^luc^* (22) [FVB.129S6(B6)-*Gt(ROSA)26Sor^tm1(Luc)Kael^*/J; The Jackson Laboratory, 005125]. Adult mice between 8 and 16 weeks of age were used for all experiments. Only female mice were used in flow cytometry analysis of R206H-FAPs. Male and female mice were used interchangeably in all other studies. Mice were genotyped by PCR and reporter fluorescence, as previously described (6, 12). All comparisons were made to littermate controls that lacked either the FOP allele or the Cre driver. These mice were phenotypically normal and are referred to as wild type throughout for simplicity.

### Skeletal muscle injury, dosing regimen, and endpoints

#### Acvr1^FLEx(R206H)/+^; CAG-Cre^ERT2^ mice.

FOP mice were randomized into treatment groups consisting of 7 mice per group or into the vehicle control group consisting of 3 animals. Injury was induced by a single intramuscular injection of 100 μL of 10 μM cardiotoxin (Sigma-Aldrich, C9759) into the gastrocnemius muscle while mice were under isoflurane anesthesia. Animals received a single 10 mg/kg dose of antibody JAB0505 by i.v. injection into the tail vein on day –1 relative to cardiotoxin injury. Serum concentrations of JAB0505 were determined by ELISA using an Alk2-Fc chimera (R&D Systems, 637-AR-100) as the capture reagent. Bound JAB0505 was detected with goat polyclonal anti–mouse IgG–HRP (R&D Systems, HAF007). Heterotopic bone formation was visualized by μCT on days 7 (*n =* 7), 14 (*n =* 7), and 20 (*n =* 4) after cardiotoxin-induced injury.

#### Acvr1^tnR206H/+^; Tie2-Cre mice.

The gastrocnemius muscle of adult mice was injured by applying 3500 to 3700 grams of force with a Randall Selitto Paw Pressure Test Apparatus (IITC Life Science). Care was taken to avoid incidental contact with the tibia and fibula, which was verified by μCT. Injuries were performed while mice were under isoflurane anesthesia. Treated mice received a single subcutaneous dose of ActA-mAb (10 mg/kg) and/or a single i.p. dose of the anti-ACVR1 monoclonal antibody JAB0505 (10 mg/kg) on the day of injury. Histological analyses were performed on days 6 and 14 after injury, flow cytometry analyses were performed on days 5 and 10 after injury, and μCT imaging was conducted on days 14, 21, 28, and 35 after injury.

To produce a subclinical injury that does not cause HO in this model, the tibialis anterior muscle was injected with 50 μL of 2.5% methylcellulose (Sigma-Aldrich) in sterile PBS. JAB0505 administration was as above. Methylcellulose-injected mice were imaged by μCT at 15 and 22 days after injury.

### μCT and HO quantification

μCT imaging of FLEx ACVR1(R206H); CAG-cre/Esr1 mice was performed using a Quantum FX μCT Cabinet X-Ray System (PerkinElmer) with mice under isoflurane anesthesia and placed in a supine position for hind limb imaging. Scan parameters included 90 kV voltage, an 80 μm voxel size, and a 4.5-minute scan time. μCT imaging of Acvr1^tnR206H/+^; Tie2-Cre mice was performed using an IVIS SpectrumCT (PerkinElmer). All μCT images were taken using medium resolution acquisition mode (75 μm voxel size; estimated radiation dose of 132 mGy; 210 second scan time) with mice under isoflurane anesthesia. The μCT images were generated and HO volumes were quantified using 3D Slicer software (http://www.slicer.org), as previously described (6, 12). Imaging of SCID hosts following cell transplantation was performed similarly.

### In vivo bioluminescence imaging

Bioluminescence images were acquired using an IVIS SpectrumCT and analyzed with Living Image 4.5 software (PerkinElmer). *Acvr1^tnR206H/+^; R26^luc/+^*; Tie2-Cre mice were injected i.p. with D-luciferin (PerkinElmer) at 150 mg/kg prior to bioluminescence imaging. The bioluminescent light emission plateau was empirically determined to be 14 to 18 minutes after D-luciferin substrate injection. Animals were anesthetized using the built-in XGI-8 Gas Anesthesia System with oxygen containing 2% isoflurane and placed into the imaging chamber. To determine bioluminescence source depth and signal intensity at the source, μCT and 2D surface radiance bioluminescence imaging was followed by 3D diffuse luminescent imaging tomography (DLIT) reconstruction as previously described (12).

### Histology and immunohistochemistry

Tissues were fixed, decalcified, and then processed for paraffin embedding as previously described (6, 12). Following deparaffinization, immunohistochemistry for ACVR1 was performed using a rabbit anti-ACVR1 antibody (Abcam, ab60157), as previously described (6). Hematoxylin, eosin, and Alcian blue staining was performed using standard methods.

### FAP isolation and culturing

Skeletal muscle tissue dissociation by enzymatic digestion and isolation of FAPs by FACS with anti-CD140a (PDGFRA)–APC (eBioscience, 17-1401-81) and anti-Ly6A/E (SCA-1)-v450 (BD Biosciences, 560653) was conducted as previously described (6, 12, 18, 35), with the following modification: magnetic depletion of CD31^+^ and CD45^+^ cells prior to FACS was omitted, and CD31^+^ and CD45^+^ cells were detected with anti-CD31-bv711 (BD Biosciences, 740690; 1:800) and anti-CD45-bv711 (BD Biosciences, 563709; 1:500) antibodies. Sorting was performed on a FACSAria II (BD Biosciences) equipped with 405, 488, 561, and 633 nm lasers.

FACS-isolated FAPs were grown on tissue culture flasks (Nunc) in DMEM (Thermo Fisher Scientific, 11995065) with 50 U/mL penicillin and 50 μg/mL streptomycin (Pen/Strep; Gibco) and 20% premium FBS (Atlanta Biologicals, lot C19032) as previously described (6, 12). FAPs were maintained at 37°C in a humidified atmosphere at 5% CO_2_. Media were changed every other day. All experiments utilized FAPs that had undergone less than 7 population doublings (approximately 3 passages).

### Quantification of cell populations by flow cytometry

All muscle groups of the posterior, lower hind limb were harvested from untreated and JAB0505-treated mice on days 5 and 10 after muscle injury, and prepared for flow cytometry as described above. For this study, FAPs were defined as CD31^–^CD45^–^SCA-1^+^ cells, the vast majority of which express the FAP marker, PDGFRα (6, 18, 36, 37). R206H-FAPs (“recombined FAPs”) were identified by recombination at the *Acvr1^tnR206H^* and *R26^NG^* loci, as negative for tdTomato-derived fluorescence and positive for GFP-derived fluorescence, respectively. Absolute cell numbers were quantified using Precision Counting Beads (BioLegend, 424902). Flow cytometry data were collected using the BD FACSAria II and was analyzed using FlowJo software (v10.6.1).

Separate experiments were conducted to quantify select immune populations within the CD45^+^ population. Tissue collection, JAB0505 dosing, injury method, analysis time points, and counting bead quantification method remained consistent with FAP quantification experiments. Injured skeletal muscle was prepared for flow cytometry in a similar manner as FAPs, with the additions of a 15-minute DNase I digestion at 37°C after initial enzymatic digestion to reduce cell clumping and potential nonspecific antibody binding, and a 1220 rpm rather than 2000 rpm centrifugation to reduce debris in the final cell preparation. TruStain FcX (anti-mouse CD16/CD32; BioLegend, 101319) was added to samples at 5 μg/mL for 10 minutes on ice, prior to antibody staining. To avoid fluorescence compensation issues due to highly overlapping emission spectra of certain fluors included in the antibody panel, we subfractionated each sample (1 biological replicate) into 2 equal volumes. The first subfraction was antibody stained for monocyte (CD45^+^CD11b^+^Ly6G^–^Ly6C^+^), macrophage (CD45^+^CD11b^+^Ly6G^–^F4/80^+^), and neutrophil (CD45^+^CD11b^+^Ly6G^+^) identification, and the second subfraction was stained for mast cell (CD45^+^CD11b^–^CD117^+^FcεRIα^+^) and T cell (CD45^+^CD11b^–^CD3^+^) identification. Since both subfractions required CD45 and CD11b detection, antibodies against both were added to the whole sample, before thorough mixing and subfractionation to mitigate potential for inconsistency in labeling of hematopoietic, myeloid, and lymphoid parent populations between subfractions of the same replicate. Fluorophore-conjugated antibodies used included anti-CD45–PerCP (BioLegend, 103129; 1:500), anti-CD11b–Alexa Fluor 700 (BioLegend, 101222; 1:500), anti-Ly6G–APC/Fire 750 (BioLegend, 127651; 1:100), anti-F4/80–Alexa Fluor 647 (BioLegend, 123121; 1:500), anti-Ly6C–bv510 (BioLegend, 128033; 1:500), anti-FcεRIα–PE-Cy7 (BioLegend, 134317; 1:750), anti-CD117–APC (eBioscience, 17-1171-81; 1:100), and anti-CD3ε–bv785 (BioLegend, 100355; 1:100). 7-AAD (eBioscience, 006993) was added to each sample at 1 μg/mL 10 minutes before flow cytometry to identify dead cells. Flow cytometry data was collected using an LSRFortessa X-20 (BD Biosciences) and was analyzed using FlowJo software (v10.6.1).

### Osteogenic and chondrogenic assays

Chondrogenic and osteogenic assays were performed using FACS-isolated and expanded FAPs, as previously described (6, 12). In brief, for chondrogenic differentiation, FAPS were resuspended at 2 × 10^7^ cells/mL and plated in 10 μL high-density micromass dots onto 35-mm tissue culture dishes (Nunc) in DMEM/F12 media with Glutamax (Thermo Fisher Scientific, 10565018) containing 5% FBS plus 1% Pen/Strep. After 10 days of culture, micromasses were fixed in 10% neutral-buffered formalin and stained with Alcian blue to detect cartilage-specific proteoglycans, as previously described (38). For osteogenic differentiation, FAPs were plated at a starting density of 3 × 10^4^ cells/cm^2^ and grown for 8 days in DMEM (Life Technologies) containing 5% FBS plus 1% Pen/Strep. Alkaline phosphatase activity was detected using a commercial kit according to the manufacturer’s recommendations (Sigma-Aldrich). For both chondrogenic and osteogenic assays, FAPs were treated continually with exogenous ligand or mAb at the indicated concentrations.

### Western blotting

Near-confluent monolayer cultures of FACS-isolated FAPs in 6-well plates were serum starved for 2 hours and then incubated for 1 hour with or without ligands and antibodies at the indicated concentrations. In the JAB0505 plus ActA-mAb condition, FAPs were incubated with ActA-mAb 10 minutes prior to, as well as during, the 1-hour incubation with JAB0505 to allow time to sequester endogenous activin A. Procedures for cell lysis, SDS-PAGE, electrophoretic transfer, antibody incubation, and washing were previously described (6). Total protein concentration was measured using the DC protein assay (Bio-Rad), and equal amounts of total protein were loaded per lane of the same gel. Antibodies used include rabbit anti–p-SMAD1/5/9 (8) (Cell Signaling Technology [CST], 13820), mouse anti–β-actin (CST, 3700), rabbit anti–mouse IgG (H+L), Alexa Fluor 647 (Thermo Fisher Scientific, A-21239) (secondary antibody for β-actin detection), and anti–rabbit IgG, HRP-linked (CST, 7074) (secondary and tertiary antibody for p-SMAD1/5/8 and β-actin detection, respectively). After the final wash, membranes were incubated in in SuperSignal West Pico PLUS Chemiluminescent Substrate (Thermo Fisher Scientific) and imaged using the IVIS SpectrumCT (PerkinElmer). p-SMAD1/5/8 and β-actin bands were imaged separately, as separate secondary antibody incubations of the same membrane were required.

### Cell transplantation and antibody treatment

FACS-isolated and expanded FAPs (1 × 10^6^) were resuspended in 50 μL of ice-cold 1× Dulbecco’s PBS (DPBS; Gibco) and injected into the pinch injured gastrocnemius muscle of SCID hairless outbred mice (SHO*-Prkdc^scid^Hr^hr^*; Charles River) as previously described (6, 12). Treated SCID host mice received a single subcutaneous dose of ActA-mAb (10 mg/kg) and/or a single i.p. dose of the anti-ACVR1 mAb, JAB0505 (10 mg/kg), on the day of injury.

### Statistics

Statistical analysis was performed using GraphPad Prism. All numerical values are presented as mean values ± SEM or SD. Two-tailed, unpaired *t* test, 2-tailed unpaired *t* test with Welch’s correction for unequal variance, 1-way ANOVA with Tukey’s multiple-comparison test, and 2-way ANOVA with Sidak’s multiple-comparison test were used to determine significance, as described in the corresponding figure legends. Differences were considered significant at a *P* value of 0.05 or less.

### Study approval

All animal studies were approved by the Institutional Animal Care and Use Committee of the University of Connecticut or Alexion Pharmaceuticals.

## Author contributions

DJG, JWH, JBLS, and SJS designed the research studies. JBLS, SJS, JTC, KB, PB, LNA, and PMD conducted the experiments and acquired the data. All authors analyzed the data. DJG, JWH, JBLS, and SJS wrote the manuscript. DJG and JWH supervised the project. JBLS, and SJS are co–first authors. JBLS assisted with project administration and is listed first in the authorship order.

## Supplementary Material

Supplemental data

## Version 1 05/03/2022

In-Press Preview

## Version 2 06/15/2022

Electronic publication
