# Supplementary material for: An anti-ACVR1 antibody exacerbates heterotopic ossification by fibro-adipogenic progenitors in fibrodysplasia ossificans progressiva mice
Source: J Clin Invest. 2022 Jun 15;132(12):e153795. doi: 10.1172/JCI153795 (PMC9197527; doi:10.1172/JCI153795)
Supplement: Supplemental data [file jci-132-153795-s063.pdf]

Supplemental Figures 1-10

A

MGWSCIILFLVATATGVHSEVQLQQSGAELVRPGASVRLSCTASGFNIKDSLMHWVKQRPE
QGLEWIGWIDPEDGETRYAPNFQDKATITAVTSSNTAYLQLSSLTSEDSAIYYCARYTSRYY
TMDYWGQGTSVTVSSAKTTPPSVYPLAPGSAAQTNSMVTLGCLVKGYFPEPVTVTWNSGS
LSSGVHTFPAVLQSDLYTLSSSVTVPSSTWPSQTVTCNVAHPASSTKVDKKIVPRDCGCKP
CICTVPEVSSVFIFPPKPKDVLTITLTPKVTCVVVDISKDDPEVQFSWFVDDVEVHTAQTKPR
EEQINSTFRSVSELPIMHQDWLNGKEFKCRVNSAAFPAPIEKTISKTKGRPKAPQVYTIPPPK
EQMAKDKVSLTCMITNFFPEDITVEWQWNGQPAENYKNTQPIMDTDGSYFVYSKLNVQKS
NWEAGNTFTCSVLHEGLHNHHTEKSLSHSPGK

B

MGWSCIILFLVATATGVHSDIQMTQSPASQSASLGESVTFTCLASQTIGTWLAWYQQKPGK
SPQLLIYAATSLADGVPSRFSGSGSGTKFSFKISSLQAEDFASYYCQQLYWTPWTFGGGTK
LEIKRADAAPTVSIFPPSSEQLTSGGASVVCFLNNFYPKDINVKWKIDGSERQNGVLNSWTD
QDSKDSTYSMSSTLTLTKDEYERHNSYTCEATHKTSTSPIVKSFNRNEC

Supplemental Figure 1. Amino acid sequence of the anti-ACVR1 antibody JAB0505. Sequence of the **(A)** heavy chains and **(B)** light chains are shown. The signal peptides are underlined.

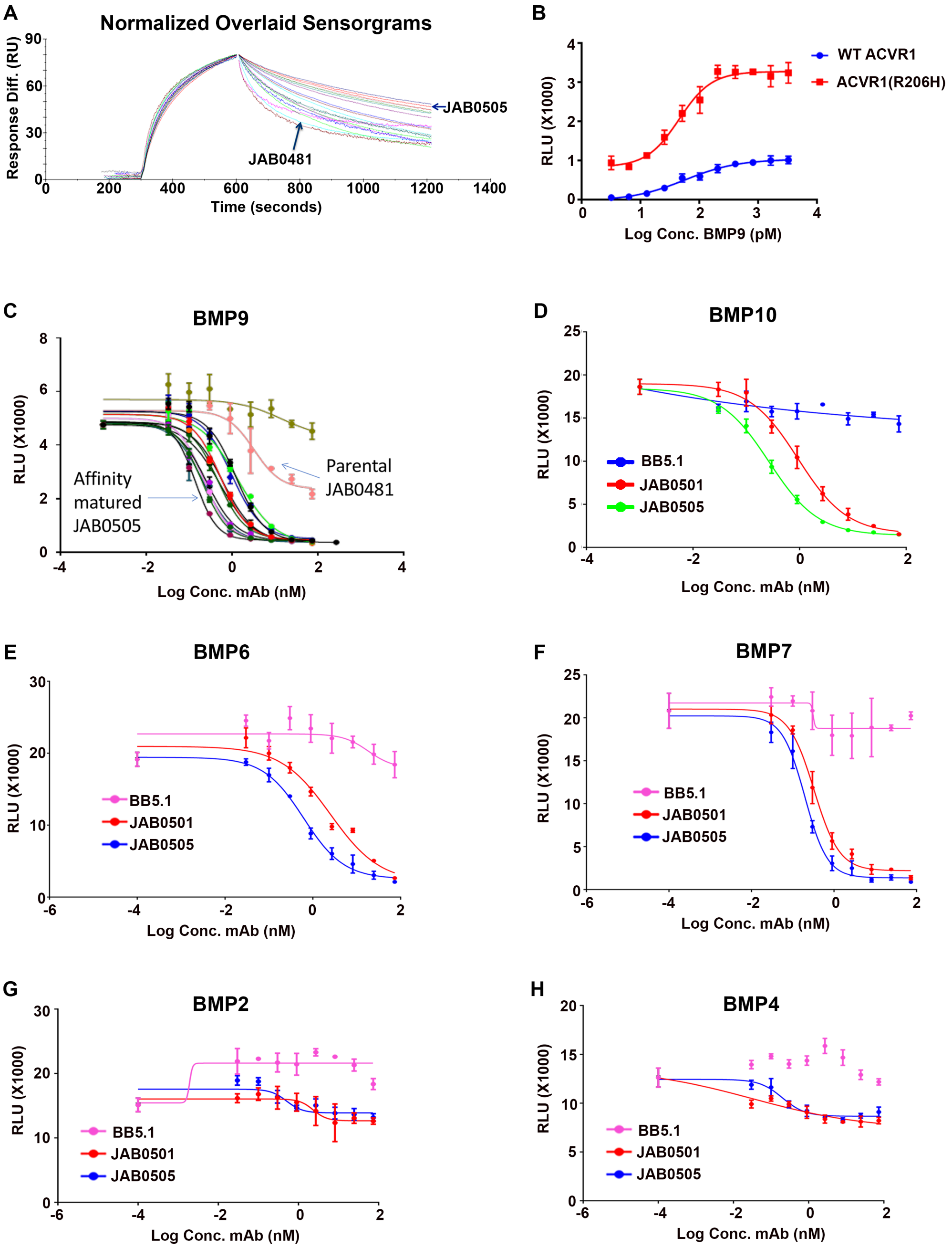

Supplemental Figure 2. Characterization of JAB0505. (A) Normalized sensorgram overlay displaying binding rate constants and equilibrium dissociation rate constants of affinity matured variants of the parental anti-ACVR1 antibody JAB0481. **(B)** Wild-type and ACVR1(R206H)-overexpressing C2C12-BRE-Luc cells were treated with BMP9 to establish a dose response. **(C)** C2C12-BRE-Luc cells were treated with BMP9 together with anti-ACVR1-antibodies. The IC50 of JAB0505 was 140 pM. **(D-H)** Anti-ACVR1 antibodies JAB0505 and JAB0501 effectively block BMP-signal activation by BMP10 (D), BMP6 (E), and BMP7 (F) in C2C12-BRE-Luc cells expressing wild-type ACVR1. Anti-ACVR1 antibodies JAB0505 and JAB0501 exhibit weak capacity to inhibit BMP2 (G) or BMP4 (H) in C2C12-BRE-Luc cells stably overexpressing ACVR1(R206H). BB5.1 is an IgG isotype control antibody. All experiments involving the inhibition of ACVR1 with anti-ACVR1 antibodies were performed in triplicate and the error bars represent ± standard deviation.

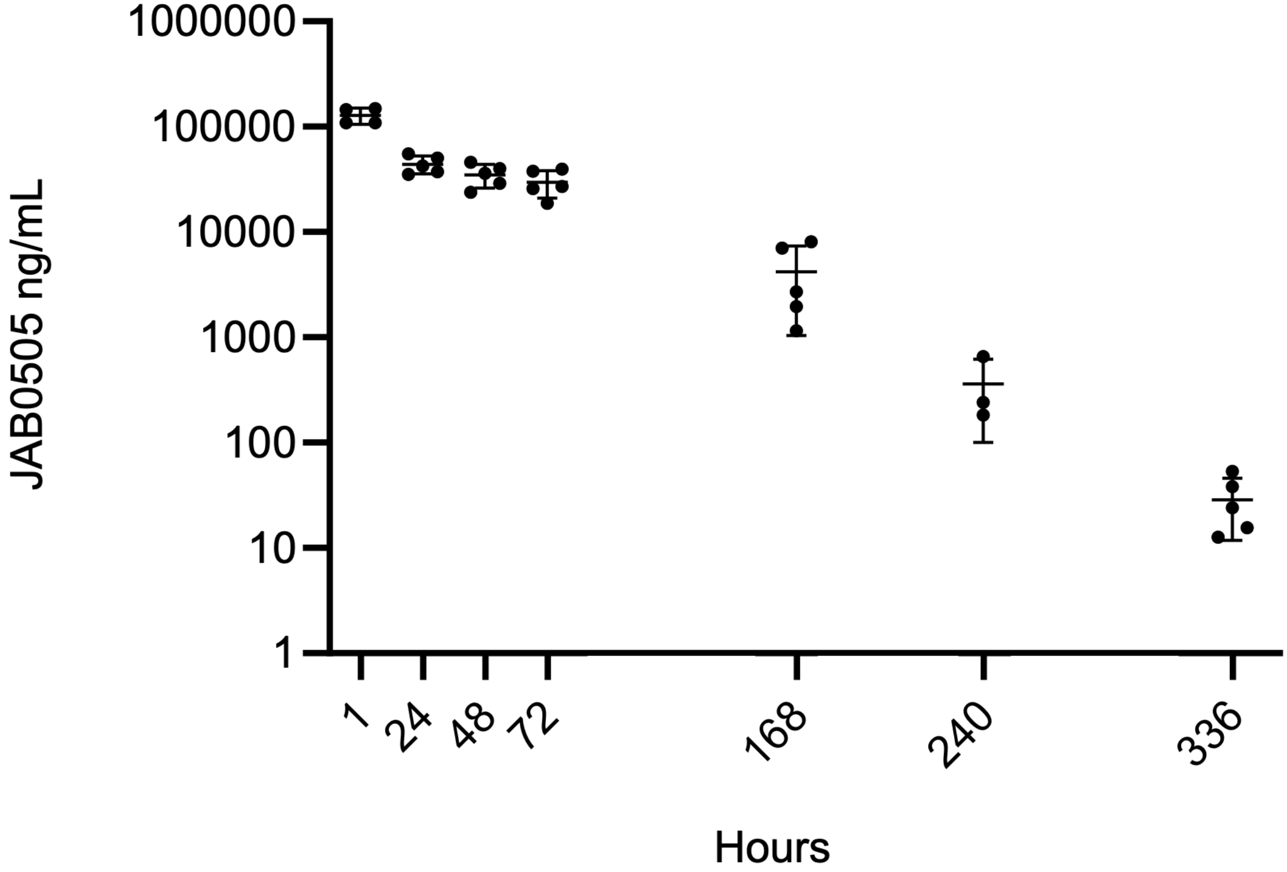

Supplemental Figure 3. Serum concentrations of JAB0505 in *Acvr1*$^{FLEx(R206H)/+}$; CAG-Cre^{ERT2} FOP mice as a function of time after administration. Filled circles represent JAB0505 concentrations from individual mice (n ≥ 3). Average concentrations are indicated as horizontal lines and the error bars represent ± standard deviation.

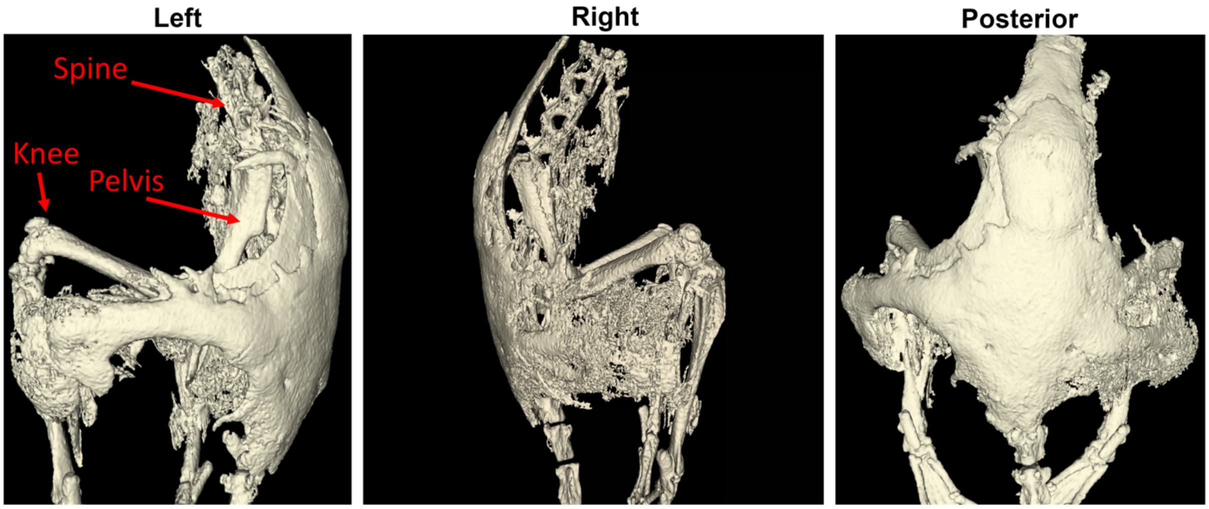

Supplemental Figure 4. An extreme case of injury-induced HO in a JAB0505-treated *Acvr1*$^{tnR206H/+}$;Tie2-Cre FOP mouse. µCT images 35 days after bilateral pinch injury of the gastrocnemius muscle. HO had aggressively spread from the distal hindlimbs to encompass the trunk.

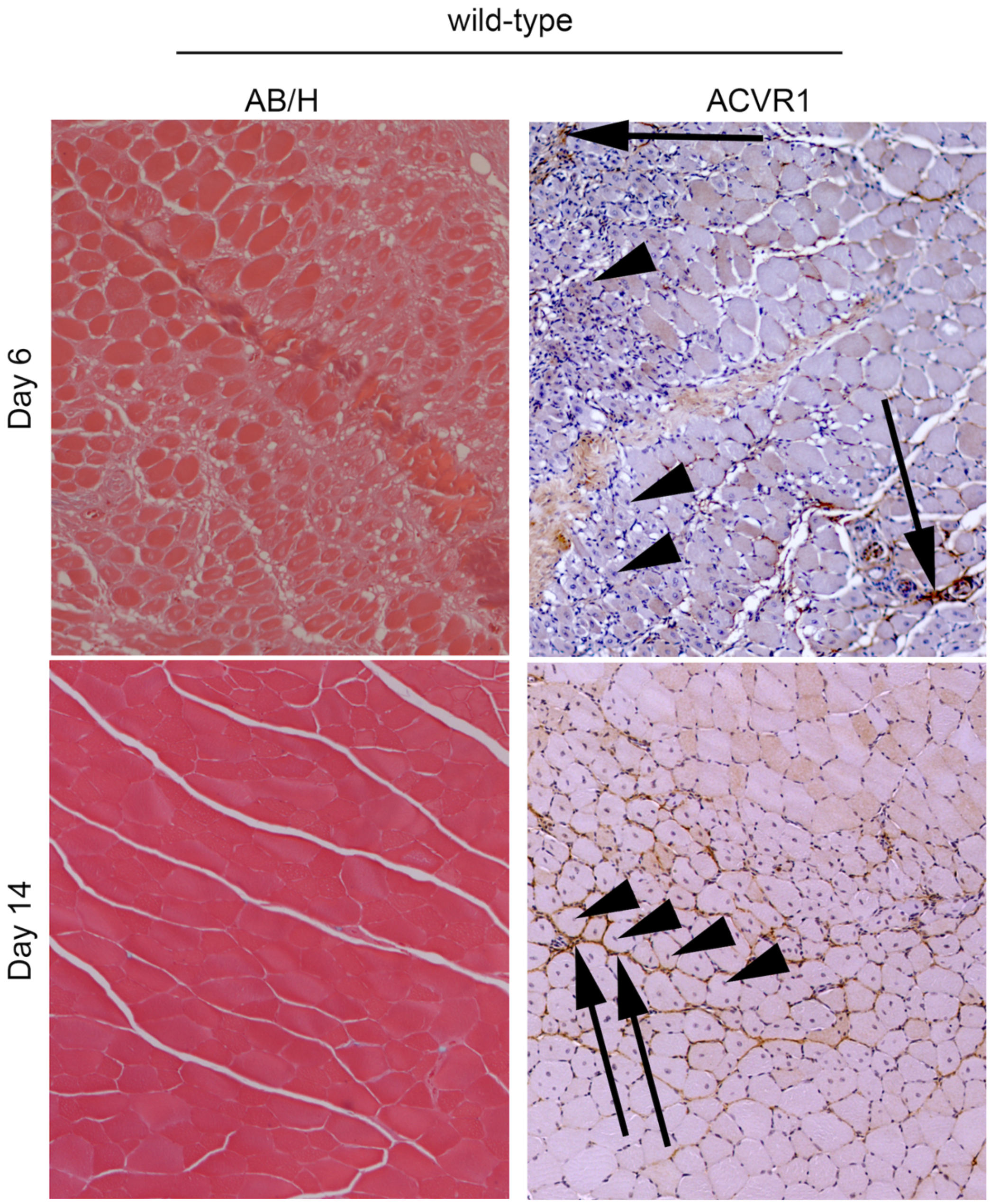

Supplemental Figure 5. Histological evaluation of muscle pinch injury in untreated wild-type mice. Alcian blue / eosin (AB/E) staining (left) and immunohistochemical staining of ACVR1 (right) on cross-sections of pinch-injured muscle at days 6 and 14 post-injury. Sections processed for immunohistochemistry were counterstained with hematoxylin. Cartilage was not detected in wild-type mice, as evidenced by an absence of alcian blue staining. At day 6 post-injury, only weak, sporadic ACVR1 staining (brown, arrows) is observed. Hematoxylin counterstaining identifies abundant centrally located myofiber nuclei (examples at arrowheads) in wild-type mice at days 6 and 14 post-injury.

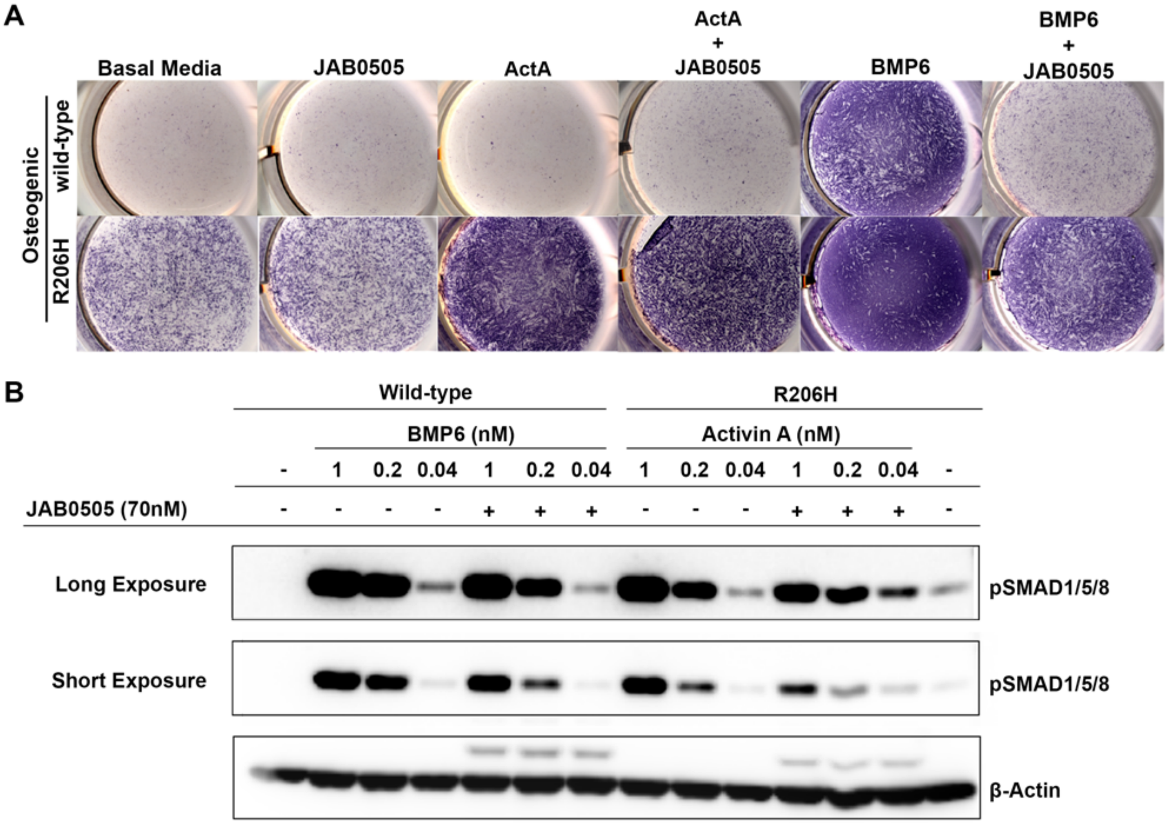

Supplemental Figure 6. Effects of JAB0505 on osteogenic differentiation and ACVR1 signal activation. (A) Osteogenic differentiation, as assessed by ALP staining (purple) and of wild-type FAPs and R206H-FAPs (R206H) cultured with or without 25 ng/mL (~1 nM) activin A or BMP6. JAB0505 was used at 10 μg/mL (~70 nM). **(B)** Western blot of SMAD1/5/8 phosphorylation (pSMAD1/5/8) in response to activin A or BMP6 with or without JAB0505. Short and long exposures for pSmad1/5/8 are provided to better show the signal dynamic range and to more accurately visualize the degree to which JAB0505 reduces pSMAD1/5/8 levels (short exposure). β-actin was used as a loading control. Faint bands above β-actin in the JAB0505-treated lanes likely reflect residual JAB0505 heavy chains.

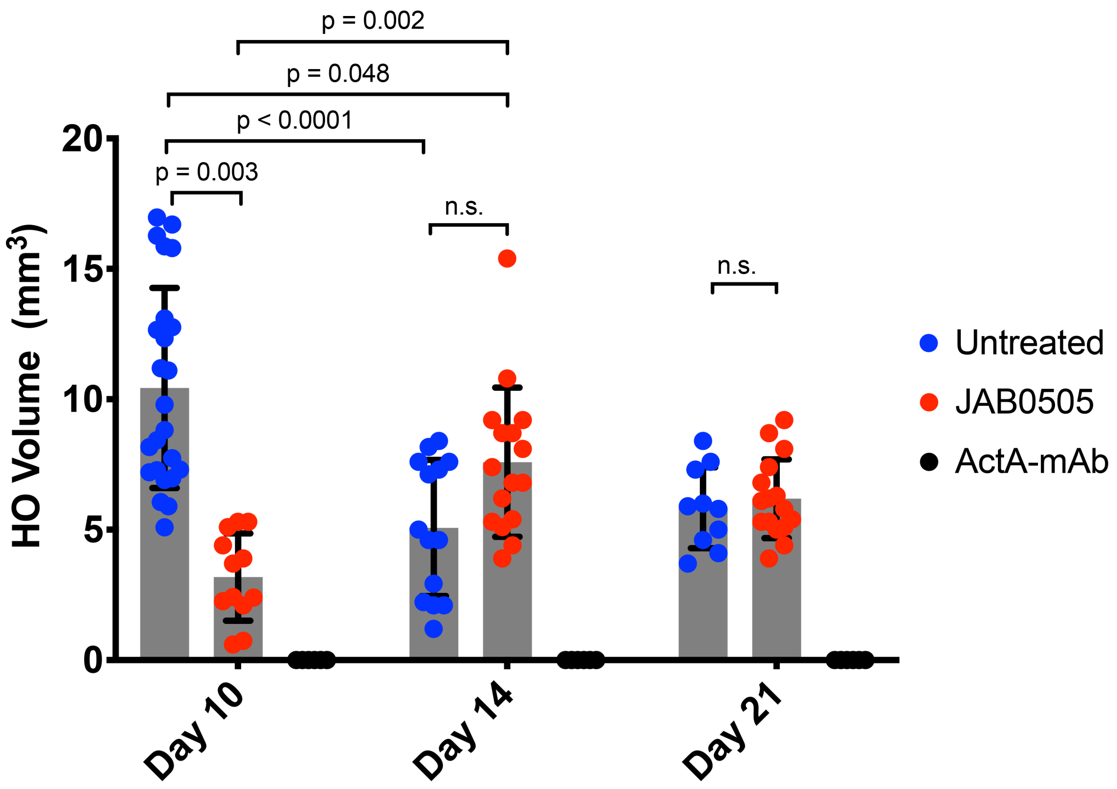

Supplemental Figure 7. JAB0505 slows the time course of HO by transplanted R206H-FAPs. Quantification of HO volume as measured by µCT of the distal hindlimb post-transplantation of R206H-FAPs into the pre-injured gastrocnemius of SCID hosts. Untreated: day 10, n = 24; day 14, n = 14; day 21, n = 10. JAB0505 (10 mg/kg): day 10, n = 12, day 14, n = 16, day 21, n = 16. ActA-mAb (10 mg/kg): n = 6 at all time points. Error bars represent ± standard deviation. Significance was determined by two-way ANOVA with Sidak's multiple-comparisons test.

.

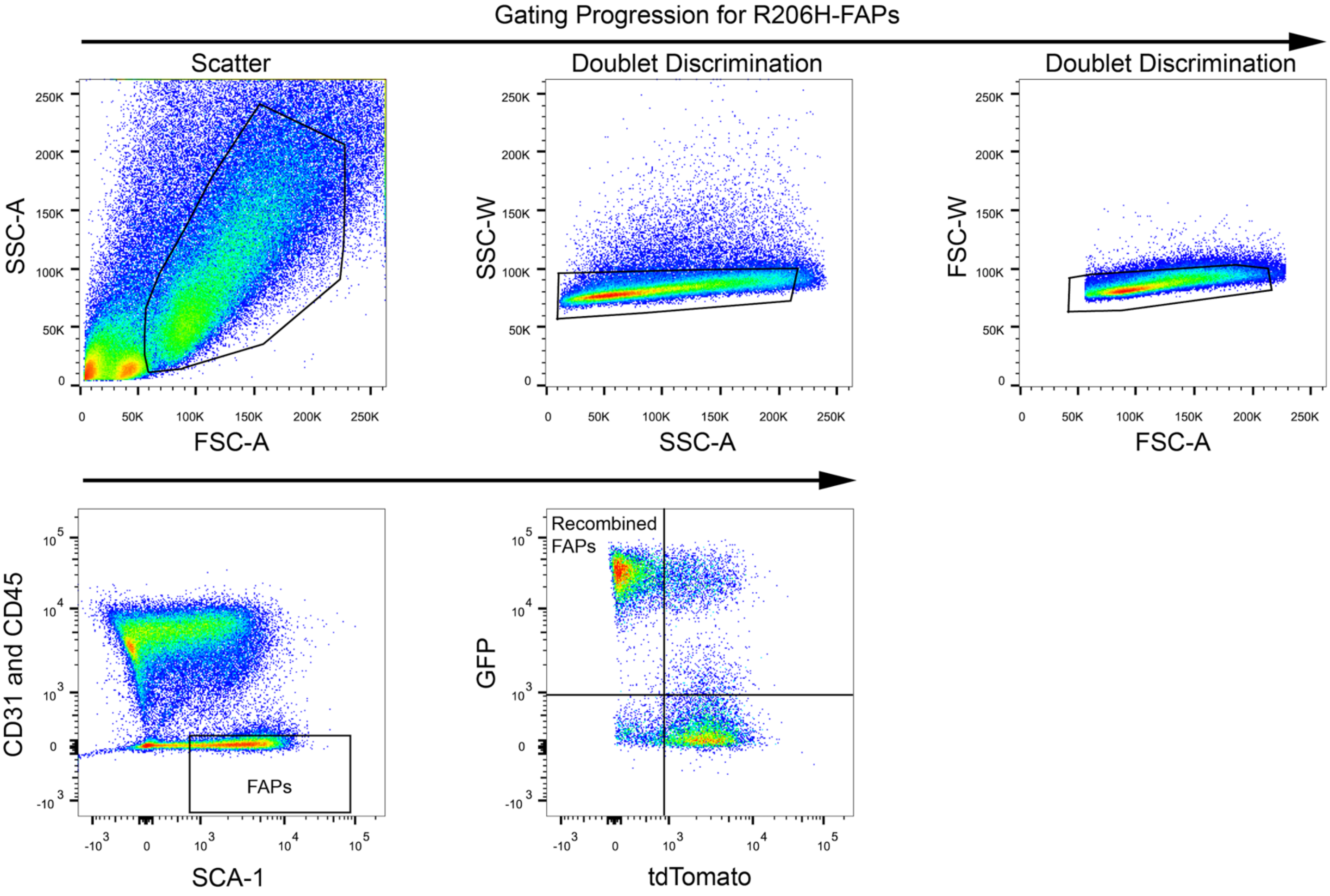

Supplemental Figure 8. FACS gating strategy for isolation and quantification of FAPs. Plots shown are representative of the gating progression used for cell population quantification and sorting of FAPs for downstream assays. Total mononuclear cells from enzymatically digested, injured skeletal muscle were gated for side/forward scatter to eliminate debris and then for doublet discrimination. When sorting FAPs, bv711 was used to gate out both CD31+ and CD45+ populations. FAPs from control muscle were identified as the CD31-CD45-SCA-1+ fraction, while recombined FAPs (R206H-FAPs) from *Acvr1*$^{R206H/+}$;Tie2-Cre muscle were identified as the CD31-CD45-SCA-1+GFP+tdTomato- fraction.

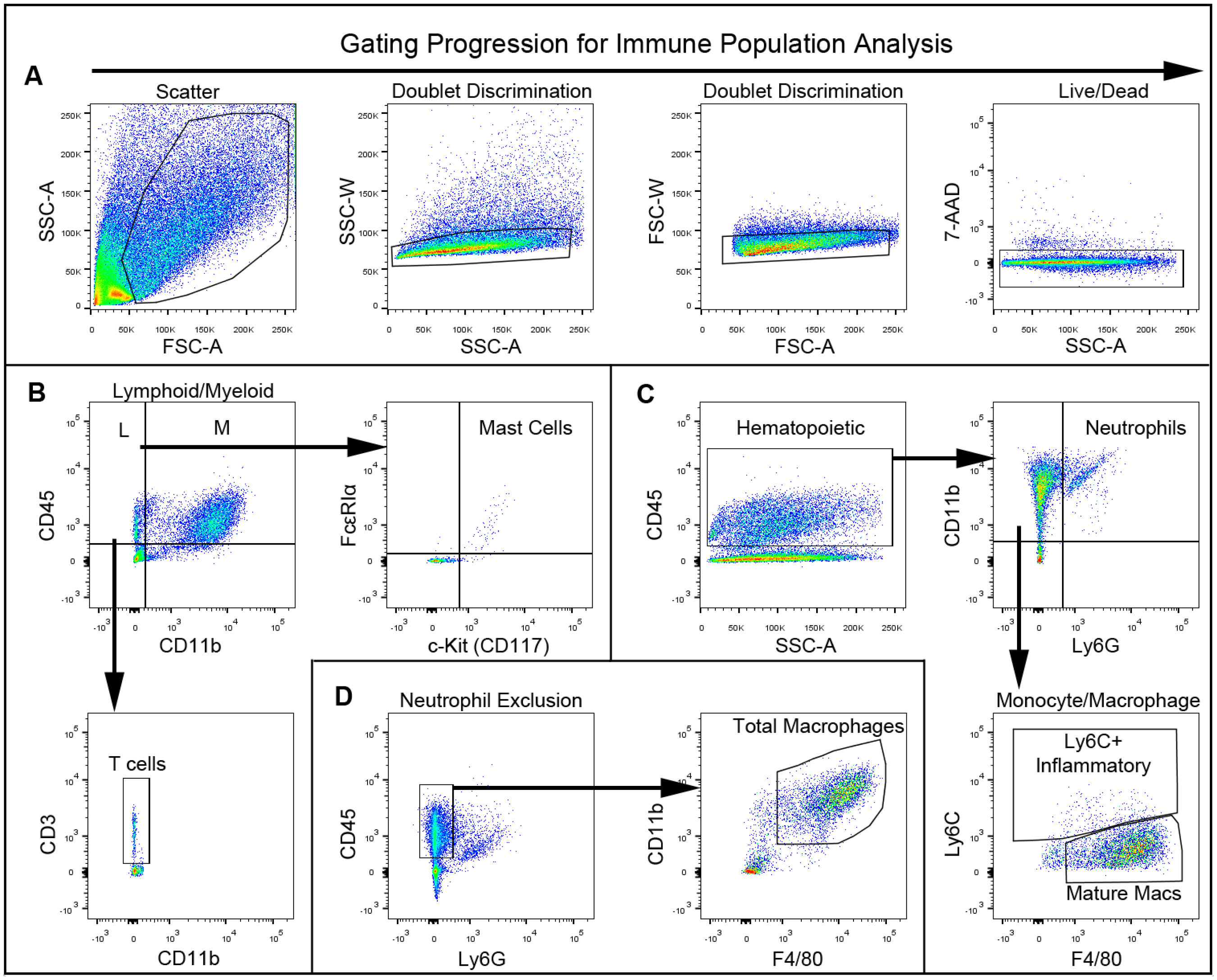

Supplemental Figure 9. Flow cytometry gating strategy for identification and quantification of relevant CD45+ immune cell populations. Plots shown are representative of the gating progression used to identify and quantify each immune population. **(A)** Total mononuclear cells isolated from injured skeletal muscle were gated for side/forward scatter to eliminate debris and doublets, and 7-AAD was used to eliminate dead cells. Equivalent sample subfractions were stained separately to avoid fluorescence compensation issues from high spectral overlap of certain fluors. Thus, after dead cell exclusion, **(B)** mast cells and T cells were identified using a separate gating progression from **(C)** monocytes, macrophages, and neutrophils. **(D)** Total macrophages were identified using a gating progression slightly modified from (C) but within the same sample subfraction. Arrows indicate gating progression.

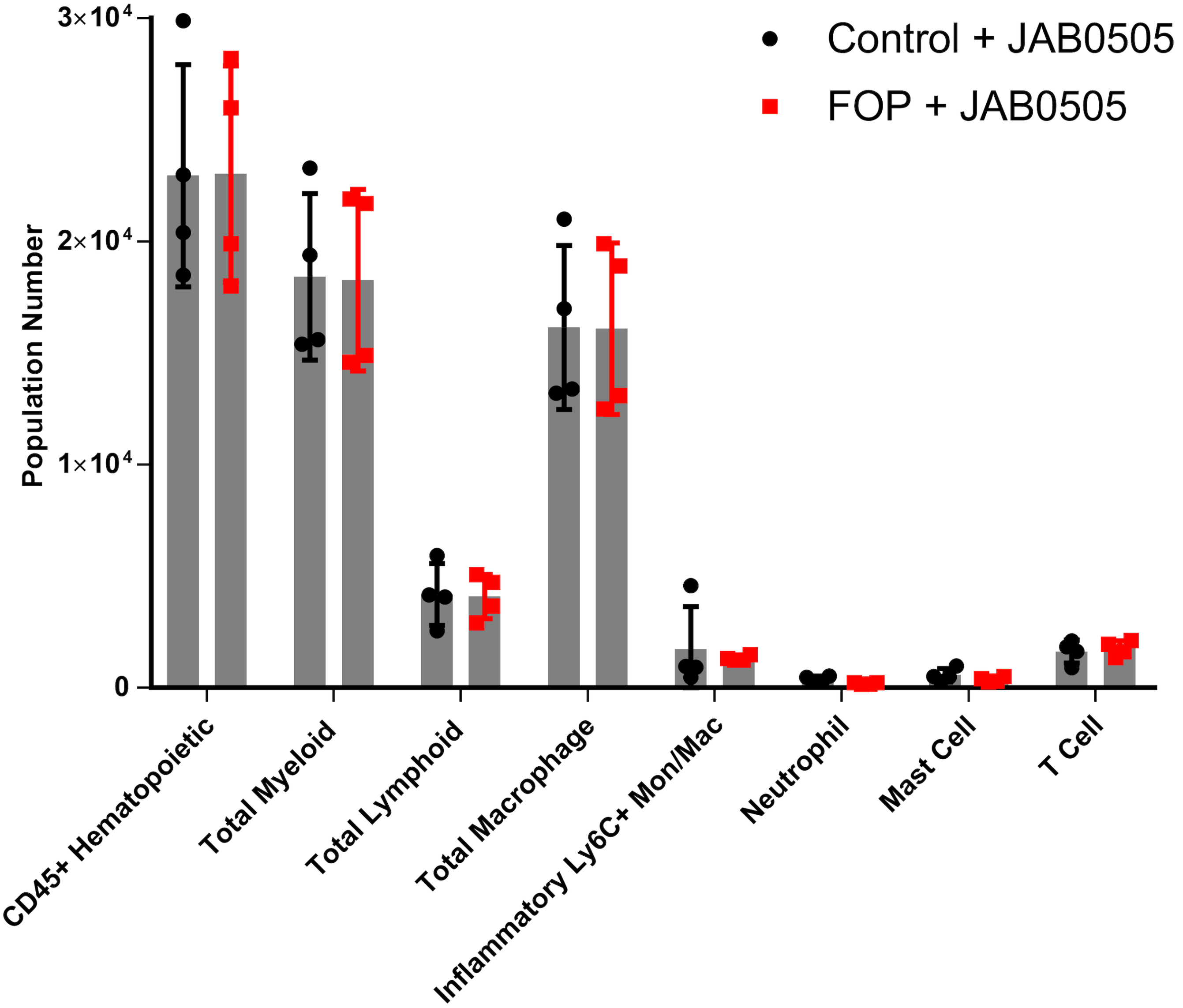

Supplemental Figure 10. JAB0505 does not increase immune population numbers in uninjured FOP muscle relative to uninjured control muscle. Flow cytometry was used to determine cell numbers of the indicated immune populations in uninjured distal hindlimb muscle at 5 days after 10 mg/kg JAB0505 was administered intraperitoneally in control (*R26*$^{NG/+}$;Tie2-Cre) and FOP (*Acvr1*$^{tnR206H/+}$;*R26*$^{NG/+}$;Tie2-Cre) mice (n = 4). Error bars represent ± standard deviation. Significance was determined using two-tailed unpaired t tests with Welch's correction for unequal variance for each immune population, with a threshold for significance of $p \leq 0.05$. No control + JAB0505 vs. FOP + JAB0505 comparisons for any of the indicated immune populations were significant.
